# Loss of SNAI1 induces cellular plasticity in invasive triple-negative breast cancer cells

**DOI:** 10.1038/s41419-022-05280-z

**Published:** 2022-09-28

**Authors:** Chrysoula Tsirigoti, Mohamad Moustafa Ali, Varun Maturi, Carl-Henrik Heldin, Aristidis Moustakas

**Affiliations:** 1 Department of Medical Biochemistry and Microbiology, Science for Life Laboratory, Uppsala University, SE-751 23 Uppsala, Sweden; 2 Department of Pharmacy, Drug Delivery, Uppsala University, SE-752 37 Uppsala, Sweden

**Keywords:** Cancer stem cells, Preclinical research

## Abstract

The transcription factor SNAI1 mediates epithelial-mesenchymal transition, fibroblast activation and controls inter-tissue migration. High SNAI1 expression characterizes metastatic triple-negative breast carcinomas, and its knockout by CRISPR/Cas9 uncovered an epithelio-mesenchymal phenotype accompanied by reduced signaling by the cytokine TGFβ. The *SNAI1* knockout cells exhibited plasticity in differentiation, drifting towards the luminal phenotype, gained stemness potential and could differentiate into acinar mammospheres in 3D culture. Loss of SNAI1 de-repressed the transcription factor FOXA1, a pioneering factor of mammary luminal progenitors. FOXA1 induced a specific gene program, including the androgen receptor (AR). Inhibiting AR via a specific antagonist regenerated the basal phenotype and blocked acinar differentiation. Thus, loss of SNAI1 in the context of triple-negative breast carcinoma cells promotes an intermediary luminal progenitor phenotype that gains differentiation plasticity based on the dual transcriptional action of FOXA1 and AR. This function of SNAI1 provides means to separate cell invasiveness from progenitor cell de-differentiation as independent cellular programs.

## Introduction

Breast cancer (BRCA), the most widespread tumor among women, is a heterogeneous disease with diverse pathological features, molecular signatures and clinical outcomes [1]. Inter-tumoral heterogeneity originates from the distinct mammary epithelial cell types that serve as the cell of origin for the accumulation of oncogenic processes [1]. Alternatively, mechanisms of de- or trans-differentiation of mammary epithelial cells eventually cause phenotypic and molecular heterogeneity in breast tumors among patients [2, 3]. Accordingly, breast cancers are classified as tumors expressing estrogen receptor-α (ERα/ESR1), progesterone receptor (PGR) and/or epidermal growth factor (EGF) family receptor HER2/ERBB2, which emanate from luminal epithelial progenitors and are classified as luminal-A or -B. Triple-negative breast cancers (TNBC) do not express the above three receptors and are subdivided into basal-like A and -B, mesenchymal (or claudin-low), mesenchymal stem-like, luminal androgen receptor and immunomodulatory [2, 4–7]. Interestingly, the majority of TNBCs, basal-like BRCA, exhibit gene expression profiles which are closely related to luminal progenitors and mammary stem cells, while oncogenic transformation of luminal cells generates tumors with basal-like characteristics [8–11].

A process of de- or trans-differentiation within the tumorigenic mammary tissue is the epithelial-mesenchymal transition (EMT) [12, 13]. Cells undergoing EMT remodel their secreted extracellular proteins and express lower numbers of E-cadherin, tight junctional components and cytokeratins, accompanied by higher numbers of N-cadherin, vimentin and certain integrins [13, 14]. A given breast tumor is composed of mixtures of epithelial, mesenchymal and intermediate phenotypes, with evidence of mesenchymal-epithelial transition (MET) [15, 16]. This heterogeneity explains the recent TNBC sub-classification explained above [5, 7, 17]. The TNBC features are reproduced well in many BRCA cell lines [18, 19], such as the mesenchymal and metastatic MDA-MB-231, originally derived from a patient pleural effusion [20]. The plasticity changes observed in breast cancer have been explained by the differential activities of signaling and transcriptional programs. A prominent signaling pathway in this respect is the transforming growth factor β (TGFβ) pathway that drives the EMT in every cell type examined so far [14]. TGFβ signaling is directly coupled to the transcriptional induction of a cohort of transcription factors that promote EMT (EMT-TFs), such as SNAI1, SNAI2, ZEB1, ZEB2, TWIST1, and TWIST2, many of which (SNAI1, ZEB1, ZEB2) associate with TGFβ signal transducers of the SMAD family and control their activity [14].

The current study focuses on the EMT-TF SNAI1, an evolutionarily conserved zinc finger transcription factor, that together with SNAI2/SLUG, forms a small family of EMT inducers during embryogenesis [21, 22]. A central function of SNAI1 is the transcriptional repression of the *E-cadherin* (*CDH1*) gene [23, 24], of tight junctional genes [25] and of the *fructose-1,6-bisphosphatase* gene that controls the rate of glycolysis [26]. Genome-wide chromatin association studies have revealed multiple genes to which SNAI1 binds in breast and colorectal cancer cells [27, 28]. In ovarian cancer cells, SNAI1 function was linked to cell-cell and cell-matrix adhesion [29]. Silencing of *SNAI1* in MDA-MB-231 cells revealed loss of invasiveness in vitro, accompanied by reduced tumor growth and metastatic potential in xenografted mice [30, 31]. *SNAI1* knockout in additional TNBC cells (HCC38 and Hs578T) indicated the generation of intermediary quasi-epithelial phenotypes, explained by the remaining and compensatory expression of SNAI2 in the same cells [28, 32]. Yet, these studies could not identify possible links between the altered TNBC phenotype and key transcription factors that define mammary epithelial cell differentiation. Loss of SNAI1 may lead to lineage plasticity that could explain some of the phenotypes in heterogeneous breast cancers. This hypothesis is examined in the present study that links *SNAI1* knockout to basal-luminal plasticity.

## Materials and methods

### Cells, reagents and treatments

Human TNBC-basal MDA‐MB‐231 cells obtained from ATCC (HTB-26, LGC Standards, UK) and Hs578T cells obtained from Dr. Paraskevi Heldin were cultured in Dulbecco’s modified Eagle’s medium (DMEM; Sigma-Aldrich AB, Stockholm, Sweden). Luminal BRCA MCF-7 and ZR-75-1 cells obtained from Dr. Paraskevi Heldin were cultured in DMEM and in Roswell Park Memorial Institute (RPMI)‐1640 (Sigma-Aldrich AB), respectively. All culture media were supplemented with 10% fetal bovine serum (FBS; Biowest, Almeco A/S, Esbjerg, Denmark), 100 U/ml penicillin and 100 μg/ml streptomycin (Sigma-Aldrich AB). All cells were kept in a humidified incubator at 37 °C and 5% CO_2_. All cell lines were frequently analyzed and found free of mycoplasma and authenticated using STR analysis. Cells were starved for 18 h in serum-free DMEM or RPMI and treated for different time periods with recombinant human TGFβ1 (PreproTech EC Ltd, London, UK) at a final concentration of 5 ng/ml, as indicated. Cells were treated with the indicated concentrations of enzalutamide (MDV3100, Selleckhem, Houston, USA) in DMEM or RPMI containing 10% charcoal-stripped FBS.

### CRISPR/Cas9 knockout

MDA-MB-231 cells were transfected with CRISPR/Cas9 and HDR plasmids targeting *SNAI1* (Santa Cruz Biotechnology Inc., CA, USA). A pool of three plasmids, each encoding one guide RNA targeting exon-1, exon-2 or exon-2/intron-2 junction, was co-transfected with the Cas9 nuclease and HDR plasmids. Two days post‐transfection, cells were selected with 4 μg/ml puromycin (Merck/Millipore, Stockholm, Sweden) and single-cell colonies were expanded. Knockout clones were validated using immunoblotting and quantitative RT-PCR to analyze the mRNA levels of each of the three SNAI1 exons.

### Transient transfection with siRNA

Cells were transfected with siRNAs (20 nM; ON-TARGETplus Non-targeting Control Pool (D-001810-10), ON-TARGETplus Human FOXA1 set of four individual siRNAs (LQ-010319-00-0002, Dharmacon/VWR, Stockholm, Sweden) using Lipofectamine RNAiMAX according to the manufacturer’s instructions (Thermo Fisher Scientific, Stockholm, Sweden).

### Plasmid transfections

*SNAI1*-KO cells were transfected using X-tremeGENE 360 transfection reagent (Merck/Millipore) with the peGFPNI SNAI1 plasmid kindly provided by Dr. Antonio Garcia de Herreros (IMIM-Hospital del Mar, Barcelona, Spain). After 48 h, cells expressing high GFP were sorted from the pool of the transfected cells by flow cytometry in 96-well plates and single-cell colonies were grown in the presence of 2.0 mg/ml geneticin (Thermo Fisher Scientific) for two weeks, generating stable SNAI1 overexpression clones.

### 3D culture of human mammospheres

Mammosphere cultures of MDA-MB-231 and ZR-75-1 cells were performed using the hanging drop method by seeding 10 000 cells per drop in complete growth medium with 20% methylcellulose for 48 h. Mammospheres were collected and resuspended in MEM-F12 (Sigma-Aldrich AB) supplemented with 25 ng/ml epidermal growth factor, 25 ng/ml basic fibroblast growth factor, 1×B27 (Thermofisher Scientific) and 2% methylcellulose (Sigma-Aldrich AB). One mammosphere was placed per well in 96-well round bottom ultra-low attachment microplates (CORNING, Wiesbaden, Germany) and cultured for 5 days. Phase-contrast pictures were acquired every 24 h for 5 days. The cross-section area of the mammospheres was calculated using the Image-J software (National Institutes of Health, Bethesda, MD, USA).

### Cell proliferation

For proliferation assessment, 50,000 MDA-MB-231 or ZR-75-1 cells per well were seeded in 6-well plates, and cell counting was performed on day five after seeding using the Luna automated cell counter (Logos Biosystems, France). The number of viable cells was used for further analysis.

### Cell viability

Cytotoxicity to decreasing serial dilutions of doxorubicin (D1515; 2 μM–7.8 nM), paclitaxel (T7402; 1 μM–3.9 nM), both from Sigma-Aldrich AB and to enzalutamide (MDV3100, Selleckhem; 50–0.1 μM) was monitored at day 2 and 5 by PrestoBlue HS reagent, following the manufacturer’s instructions (Thermo Fisher Scientific). Dimethyl-sulfoxide (DMSO) served as a vehicle. Cells treated with enzalutamide were cultured in charcoal-stripped FBS.

### Extreme limiting dilution assay (ELDA)

Cells were seeded in low-attachment 96-well plates (CORNING) in decreasing serial dilutions (100-1 cells/well), in 200 μl of serum-free MEM-F12 (Sigma-Aldrich AB) supplemented with 25 ng/ml epidermal growth factor, 25 ng/ml basic fibroblast growth factor, 1×B27 (Thermofisher Scientific). Six technical replicates for each cell plating density were created. On day 10, the number of wells containing spheres was recorded and analyzed using the online ELDA analysis program (http://bioinf.wehi.edu.au/software/elda) [33].

### Invasion of mammosphere cells

Mammospheres of MDA-MB-231 and ZR-75-1 cells generated as described above, were collected after 48 h and resuspended in a collagen I solution (1.7 mg/ml, PureCol, Advanced BioMatrix, Inc., San Diego, CA, USA) in DMEM or RPMI. One mammosphere was embedded per well, and phase-contrast images were acquired at the onset of embedding and after 24 and 48 h. Invasive growth was calculated as the area occupied by cells between the invaded area and the core mammosphere using Image-J (National Institutes of Health).

### Zebrafish extravasation assay

Staging and embryo production of Tg(Fli1:EGFP) zebrafish (*Danio rerio*), whose vasculature is marked in green, were conducted as described [34] and embryos were maintained at 34 °C. Empirical determination of sample size that provided power for discrimination between conditions was used and for this reason 200 embryos were injected per condition in order to reach a final number of viable embryos of more than 100. No randomization method was applied and the microinjector was blinded to the groups of injected cancer cells. MDA-MB-231-WT and *SNAI1*-KO cells were stained with 4 ng/μl CM-Dil Dye (ThermoFisher Scientific) for 30 min at 37 °C. At 48 h post-fertilization, approximately 400 CM-Dil Dye-labeled cells were loaded into borosilicate glass capillary needles (1 mm O.D. × 0.78 mm I.D, Harvard Apparatus, Holliston, MA, USA), and were injected into the duct of Cuvier of dechorionized embryos, anesthetized with 0.003% tricaine (Sigma-Aldrich AB), and mounted on a 10-cm Petri dish coated with 1% agarose gel, using a Pneumatic Picopump and a manipulator (WPI, Stevenage, UK). Based on pre-established criteria, only microscopy-verified, correctly injected and viable zebrafish were retained at 34 °C and imaged automatically (ImageXpress Nano, Molecular Devices, USA) 24 h post-implantation; cells extravasated from the circulation at the posterior part of 150 zebrafish per cell line were counted.

### Immunoblotting

Total cellular proteins were extracted in lysis buffer (20 mM Tris-HCl, pH 7.5, 1% nonidet P‐40, 150 mM NaCl) supplemented with a complete protease inhibitor cocktail (Roche Diagnostics Scandinavia AB, Bromma, Sweden). The lysates were sonicated for 5 min at 4 °C (30 sec ON/30 sec OFF), cleared by centrifugation, and protein concentration was measured by Bradford assay (Sigma-Aldrich AB). Equal amounts of protein (40 μg) were subjected to sodium dodecyl sulfate polyacrylamide gel electrophoresis and transferred to a PVDF membrane using a Bio-Rad wet transfer unit (Bio-Rad Laboratories Inc., Sundbyberg, Sweden). Filters blocked with 5% bovine serum albumin (BSA; Saveen Werner, Limhamn, Sweden) were incubated with primary antibodies and horseradish peroxidase-conjugated anti-mouse or anti-rabbit secondary antibodies (Thermofisher Scientific) as listed in Table S7, followed by enhanced chemiluminescence assays using the Millipore kit (Merck/Millipore). The original, uncropped immunoblots are listed as the last Supplementary figure.

### Immunofluorescence microscopy

MDA-MB-231 and ZR-75-1 cells fixed in 3.7% formaldehyde for 15 min, were incubated in 0.1 M glycine for 45 min and permeabilized in 0.5% Triton X-100 for 10 min, blocked in 5% BSA/PBS for one h at room temperature and incubated with primary antibodies (Table S7) in 1% BSA/PBS overnight at 4 °C. Samples were incubated with Alexa-Fluor-488 or Alexa-Fluor-546 secondary antibodies (Thermofisher Scientific) at a dilution of 1:500 in PBS for 1 h at room temperature. Phalloidin (1:500 in PBS) staining lasted for either 1 h at room temperature (2D) or overnight at 4 °C (3D). The nuclei were stained afterward with 4′,6-diamidino-2-phenylindole (DAPI, Sigma-Aldrich AB) at a dilution 1:1000 in PBS for 10 min at room temperature. Coverslips mounted with Fluoromount-G (Southern Biotech, AH Diagnostics, Solna, Sweden) were examined by a Nikon Eclipse 90i fluorescence microscope (Nikon Corp., Tokyo, Japan) or a Zeiss LSM700 confocal microscope (Carl Zeiss AB, Stockholm, Sweden). Five to 6 random pictures were taken with a 10× or 20× objective at the same exposure time.

### Chromatin immunoprecipitation

Cells were harvested (20 × 10^6^) by trypsinization followed by PBS wash at room temperature and crosslinked using 10 ml of 1% formaldehyde (dissolved in PBS) for 10 min at room temperature and kept on gentle shaking. The reaction was quenched with 0.125 M final glycine concentration for 5 min, followed by centrifugation at 1000 × *g* at 4 °C for 10 min. The crosslinked pellets were washed once with cold PBS followed by a centrifugation step at 1000 × *g* at 4 °C for 10 min. The cell pellet was resuspended in 1 ml of SDS lysis buffer (0.1% SDS, 0.5% Triton X-100, 20 mM Tris-HCl, pH 8, and 150 mM NaCl, 1 mM PMSF, and 1× protease inhibitors) and incubated on ice for 30 min with continuous pipetting. Then, the chromatin of the lysed pellet was sheared using a Bioruptor for a total of 30 cycles (30 s ON, 30 s OFF at high pulse). The insoluble components were removed by centrifugation at 13,000 × *g* at 4 °C for 10 min, while 5% of the total lysate served as input. Cleared lysates were incubated with the respective antibodies (FOXA1 or IgG) for immune precipitation overnight at 4 °C. An aliquot of 4 μg of antibody was used per 1 mg of lysate protein. We allowed the immune complexes to bind to Dynabeads Protein A (ThermoFisher Scientific) for 3 h at 4 °C with gentle rotation and then the immune complexes bound to beads were separated using magnetic precipitation followed by two washes of 750 µl low salt buffer (0.1% SDS, 1% Triton-X 100, 2 mM EDTA, 20 mM Tris-HCl, pH 8, 150 mM NaCl, 0.5 mM PMSF and 1× protease inhibitors) for 5 min at 4 °C with gentle rotation. The immune complexes were then washed with 750 µl of high salt buffer (0.1% SDS, 1% Triton-X 100, 2 mM EDTA, 20 mM Tris-HCl, pH 8, 500 mM NaCl, 0.5 mM PMSF and 1× protease inhibitors) for 5 min at 4 °C with gentle rotation. To elute immune-precipitated material from the beads, we added 400 μl of elution buffer (50 mM Tris-HCl, pH 8, 20% SDS, 10 mM EDTA and 0.5 mM PMSF) and incubated at 55 °C for 30 min with agitation. A de-crosslinking step was performed by incubating the beads with 300 µl of proteinase K buffer (100 mM NaCl, 10 mM Tris-HCl pH 7, 0.5% SDS, 10 µg/ml Proteinase K) at 65 °C overnight. DNA isolation was performed using the QIAquick PCR purification kit (QIAGEN). Sequences of oligonucleotide primers used in ChIP assays are listed in Table S8. The data were plotted as fold-enrichment of the specific ChIP signal relative to the IgG control and presented as average values with standard deviations of four biological replicates, each with triplicate technical repeats.

### RNA analysis

One μg of total RNA extracted with Nucleospin RNA-plus Kit (Macherey-Nagel, AH Diagnostics, Solna, Sweden) and quantified on a NanoDrop 2000 (Thermofisher Scientific) was reverse transcribed using the iScript cDNA synthesis kit (Bio-Rad Laboratories Inc.) according to the manufacturer’s protocol. Sequences of oligonucleotide primers used in RT-qPCR assays are listed in Table S8. Specific target gene expression was normalized to the reference gene *GAPDH*.

### Real-time PCR analysis

Real-time PCR of cDNA or chromatin DNA (ChIP analysis) samples was performed on a Bio-Rad CFX96 cycler (Bio-Rad Laboratories Inc.) using the qPCRBIO SyGreen 2× Master Mix (PCR Biosystems, London, UK) and primers (Table S8). Specific amplicon numbers were normalized to a reference gene (usually *GAPDH*). DNA amplification was calculated based on the ΔΔC_t_ method and plotted as average values with standard deviations of at least three biological replicates, each with triplicate technical repeats.

### RNA-seq analysis

Five hundred nanograms total RNA extracted using ReliaPrep™ RNA Cell Miniprep System (Promega, Madison, WI, USA), were checked using the Agilent-2100 Bioanalyzer System and subjected to library preparation utilizing the TruSeq stranded total RNA Gold library preparation kit with RiboZero Gold treatment and unique dual indexes according to the manufacturer’s instructions (Protocol # 1000000040499, Illumina Inc., San Diego, CA, USA). The paired-end reads of 150 bp were generated in an S4 flowcell with v1.5 sequencing chemistry on a NovaSeq-6 000 platform (Illumina Inc.). Read quality was investigated by FastQC tool v0.11.9, adapters and low-quality reads were trimmed by trimmomatic tool v0.36 [35], and trimmed reads were aligned against the reference human genome (GRCh38) using STAR aligner v2.7.2b with two-pass mode [36]. After checking alignment quality by SAMStat tool v1.5.1 [37], high-quality (Q = 30) aligned reads were annotated and quantified against the gencode comprehensive gene annotation release 38 (GRCh38.p13) using featureCounts of subread package v2.0.0 [38]. Differential gene expression analysis was performed by DESeq2 Bioconductor package in R [39], thus calculating normalized counts per million reads. We considered cut-off criteria of log_2_ fold-change ±2 and false discovery rate (FDR) < 0.05 for differential expression, visualized in RStudio v1.4.1717 with R v4.0.5. All primary RNA-sequencing data are available at GEO (accession number GSE210870; https://www.ncbi.nlm.nih.gov/geo/query/acc.cgi?acc=GSE210870).

### Pathway-enrichment, network analysis and ChIP-sequencing annotation

We investigated enriched hallmarks and biological processes based on transcriptome profiles utilizing the gene set enrichment analysis (GSEA) tool and the molecular signature database MSigDBv6 [40, 41]. Pathway- and transcription factor-enrichment analysis of differentially expressed genes was based on the g:Profiler package in R [42], and network analysis on EnrichmentMap plugin-v3.3.3 [43] and visualization via Cytoscape-v3.8.2 [44]. We annotated ChIP-sequencing BED files using the annotatePeaks.pl program integrated into HOMER v4.11 suite [45].

### TCGA and Cancer Cell Line Encyclopedia (CCLE) data analysis

The log_2_ transformed transcript per kilobase million (TPM) values of the queried genes were obtained from the Pan-Cancer Atlas analysis of the Cancer Genome Atlas (TCGA) [46] available at cBioportal [47]. We examined the expression distribution of the queried genes in different subtypes and compared the median expression difference using the Wilcoxon rank-sum test (*U* test). Multinomial logistic regression analysis was based on the neural network-based nnet package in R. TCGA samples (*n* = 945) formed a training (*n* = 609) and a validation set (*n* = 336), before testing the multivariate model in the training set, and investigating the predictive power of the model in the validation set. Statistical significance of the variables was based on a two-tailed *Z* test and a confusion matrix assessed performance of the model. For binomial logistic regression analysis, we classified TCGA samples into basal and non-basal tumors, and implemented a generalized linear model to fit the data in R. Statistical significance of the variables was assessed by ANOVA testing, and performance of the predictive model by ROCR package in R [48]. For cell line data, we downloaded TPM pseudo-counts of 61 BRCA cell lines with the metadata from the CCLE [49], available at the depmap portal [50]. For the compatibility with CCLE data, we re-aligned and calculated the log_2_ pseudo-counts TPM of MDA-MB231-WT and *SNAI1*-KO cells using the RSEM tool [51]. Heatmap visualization and hierarchical clustering were done using the hclust algorithm in pheatmap package in R.

### Survival analysis

Relapse-free survival (RFS) and distant metastasis-free survival (DMFS) in basal BRCA patients were predicted using the KM plotter database [52]. Samples were stratified, compared using auto-select cut-off and *p*-value was calculated by log-Rank test.

### Data analysis and statistics

Results express mean values of at least three independent experiments (biological repeats) as explained in the figures. The precise number of replicates is indicated in every figure legend. The number of technical and biological replications was determined by the efficiency of the cell-based assay used. Error bars represent standard error of the mean (SEM). We selected the appropriate statistical method based on sample content and variation within each group of data that was compared. The variance was similar between the groups that have been compared. Accordingly, two-group comparisons were performed using either two-tailed unpaired Student’s *t*-test or Wilcoxon rank-sum test (*U* test). Statistical significance is represented by *p*-values *p ≤ 0.05, **p ≤ 0.01, ***p ≤ 0.001. Additional statistical methods are described in the previous method sections.

### Reporting summary

Further information on research design is available in the Nature Research Reporting Summary linked to this article.

## Results

### TNBC-basal cells with mesenchymal features express high *SNAI1* levels

**Fig. 1:**
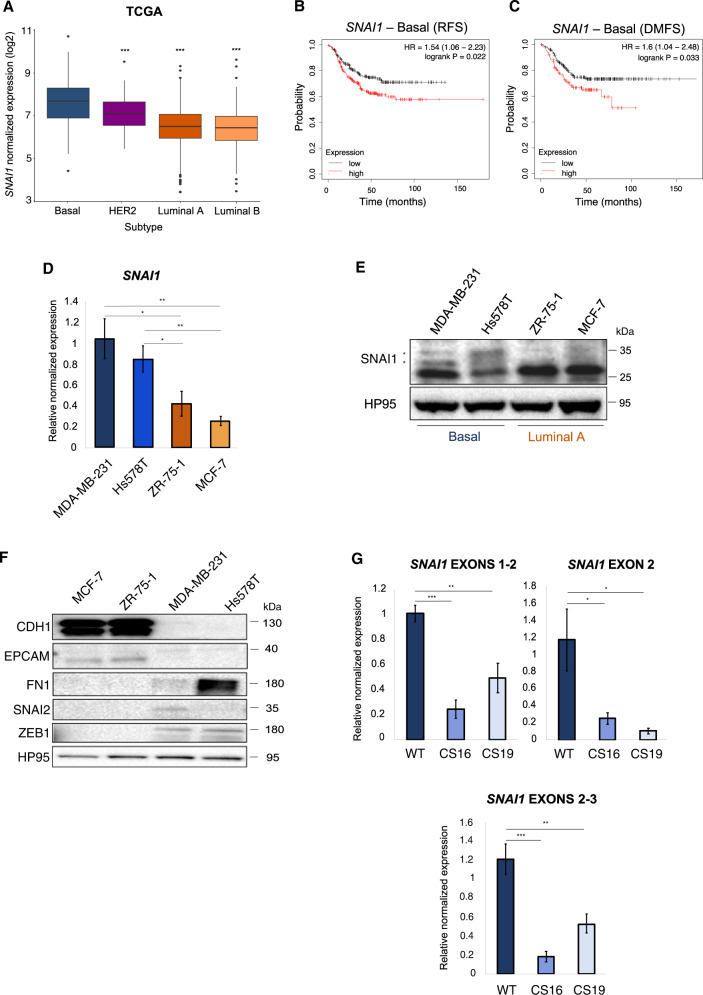
Basal BRCA cells express high levels of SNAI1 and are highly mesenchymal. **A** Expression of *SNAI1* in various subtypes of human BRCA retrieved from TCGA datasets (Basal *n* = 171, HER2 *n* = 78, Luminal A *n* = 499, Luminal B *n* = 197). The basal subtype was used as a reference to estimate the difference in *SNAI1* expression using Wilcoxon rank-sum test (*U* test). Higher *SNAI1* levels correlate with shorter relapse-free survival (RFS, *n* = 392) (**B**) and distant metastasis-free survival (DMFS, *n* = 306) (**C**) in human basal BRCA patients. Basal BRCA samples were stratified and compared in the KM plotter database using auto select cut-off. **D** RT-qPCR analysis of *SNAI1* mRNA levels in MDA-MB-231 and Hs578T (basal), ZR-75-1 and MCF-7 (luminal A) BRCA cells. Values represent fold-change of mRNA expression normalized to *GAPDH* and expressed relative to the level in MDA-MB-231 cells. **E** Protein expression levels of SNAI1 in MDA-MB-231 and Hs578T (basal), ZR-75-1 and MCF-7 (luminal A) BRCA cells. HP95 serves as loading control. Stars indicate the protein bands that are specific for SNAI1. **F** Protein expression levels of the epithelial markers CDH1, EPCAM, of the mesenchymal marker FN1, and of the EMT-TFs SNAI2 and ZEB1 in MDA-MB-231, Hs578T, ZR-75-1 and MCF-7 cells. **G** RT-qPCR analysis of mRNA levels for each of the three *SNAI1* exons in MDA-MB-231-WT and *SNAI1*-KO cells. Values represent relative mRNA expression normalized to *GAPDH*. Data in **D** are presented as mean values of three biological replicates ± SEM and in **G** as mean values of five biological replicates ± SEM, each in technical triplicates and *p*-values are shown based on two-tailed unpaired Student’s *t*-test. **E** and **F** show representative immunoblots of three independent biological replicates along with molecular mass markers in kDa. *p*-values *p ≤ 0.05, **p ≤ 0.01, ***p ≤ 0.001.

Analysis of the BRCA gene expression dataset from TCGA [46, 47] indicated a significant upregulation of *SNAI1* expression in the TNBC-basal subtype, compared to other subtypes (Fig. 1A). Data provided by the CCLE confirmed that basal-B cells expressed significantly higher levels of *SNAI1* compared to HER2 and luminal cells (Supplementary Fig. 1A, B), while elevated *SNAI1* levels predicted shorter relapse-free survival (RFS) and distant metastasis-free survival (DMFS) among patients with basal BRCA (Fig. 1B, C). Validation revealed elevated SNAI1 mRNA and protein expression in basal (MDA-MB-231, Hs578T) over luminal (ZR-75-1, MCF-7) cell lines (Fig. 1D, E). EMT-basal subtypes upregulate the expression of different EMT-TFs in addition to SNAI1 [5, 53, 54]. Accordingly, epithelial (CDH1, EpCAM), EMT-TF SNAI2/SLUG and ZEB1, and mesenchymal (fibronectin/FN1) protein analysis demonstrated high EMT-TF expression accompanied by decreased epithelial protein expression in basal relative to luminal cell lines; luminal cell lines inversely lacked EMT-TF expression, and expressed CDH1 and EpCAM (Fig. 1F).

To determine the role of SNAI1 in TNBC-basal cells, we stably ablated the *SNAI1* gene in MDA-MB-231 (this study) and Hs578T [28] cells using CRISPR/Cas9 technology (Supplementary Fig. 1C). For further characterization, we selected two stable cell clones (CS16, CS19), which exhibited downregulated mRNA expression at all three *SNAI1* exons (Fig. 1G), and no detectable SNAI1 protein (Supplementary Fig. 1D), in the absence or presence of TGFβ1, the potent inducer of SNAI1 expression [23].

### *SNAI1* knockout induces partial MET

To understand downstream signaling pathways dependent on the function of *SNAI1*, we analyzed the transcriptomic profiles of MDA-MB-231-WT and *SNAI1*-KO cells. Alignment of RNA-sequencing reads against the reference genome confirmed the genetic rearrangement of the *SNAI1* locus in KO cells, represented by *SNAI1* deletions at the 5’ end of exon-2 and of intron-2 (Supplementary Fig. 1E). No alterations in the closely related *SNAI2* (*SLUG*) gene were observed (Supplementary Fig. 1F), confirming that the knockout was specific for *SNAI1* without affecting *SNAI2*.

**Fig. 2:**
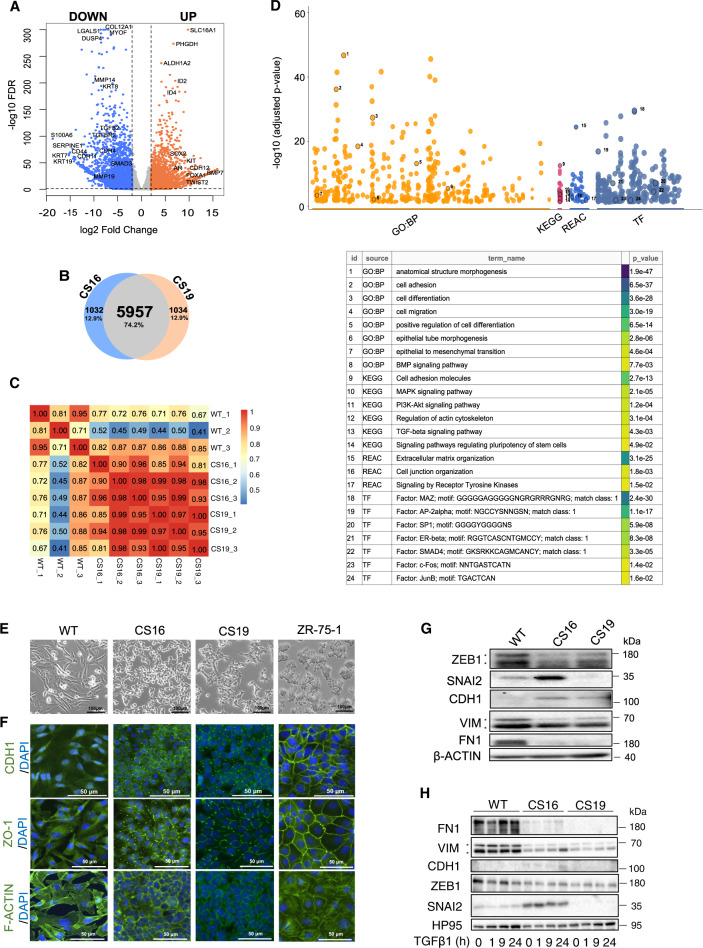
*SNAI1* knockout generates an intermediate mesenchymal to epithelial phenotype. **A** Volcano plot depicting the up- and down-regulated genes in MDA-MB-231 *SNAI1*-KO cells relative to WT cells. The horizontal dashed line represents the statistical significance threshold (FDR < 0.05) and the vertical lines indicate the expression fold-change threshold (−2 ≤ log_2_ ≥ 2). **B** Venn diagram showing the number of differentially expressed genes in *SNAI1*-KO CS16 and CS19 clones relative to WT cells. **C** Heatmap indicating the correlation coefficients among MDA-MB-231-WT, CS16 and CS19 *SNAI1-*KOs based on whole transcriptome profiling. **D** Manhattan plot (upper panel) illustrating the pathway-enrichment analysis of the 5957 differentially expressed genes in *SNAI* KOs retrieved from different databases. Twenty-four GO terms are circled and numbered in the plot and listed in detail in the table. GO:BP, gene ontology biological processes; REAC, Reactome; TF, transcription factors. The *p*-values are adjusted for multiple testing and are also color-coded. **E** Phase-contrast images showing cell morphology in MDA-MB-231-WT and *SNAI1*-KO cells. Scale bars = 100 μm. **F** Representative immunofluorescence staining pictures of CDH1, ZO1, and F-actin (green) in MDA-MB-231-WT, *SNAI1*-KOs and ZR-75-1. Nuclei are stained in blue with DAPI. Scale bars = 50 μm. **G** Protein expression levels of the EMT transcription factors ZEB1, SNAI2, the mesenchymal proteins FN1, VIM and the epithelial protein CDH1 in MDA-MB-231-WT and *SNAI1*-KO cells. **H** Protein expression levels of the aforementioned proteins in MDA-MB-231-WT and *SNAI1*-KO cells stimulated with 5 ng/ml of TGFβ1 for the indicated time periods. HP95 serves as loading control. **E** and **F** show representative photomicrographs from experiments performed in three independent biological replicates. **G** and **H** show representative immunoblots of three independent biological replicates along with molecular mass markers in kDa.

Differential gene expression analysis of WT and *SNAI1*-KO cells showed upregulation of 3437 and downregulation of 4387 genes ((log_2_ fold-change ±2 and FDR < 0.05; Fig. 2A, Supplementary Table 1). CS16 and CS19 cells shared 5957 differentially expressed genes and presented a strong correlation (Fig. 2B, C, Supplementary Table 2). Pathway-enrichment analysis of the common genes in *SNAI1*-KOs revealed that EMT and TGFβ pathways were among the most significantly downregulated molecular signatures (Supplementary Fig. 2A, B, Supplementary Table 3). Additional biological processes significantly modulated by the knockout included cell adhesion, migration, differentiation, extracellular matrix organization as well as KRAS and WNT/β-catenin pathways (Fig. 2D, Supplementary Fig. 2A, B). Hierarchical clustering based on the expression profiles of 18,817 genes in 61 BRCA cell lines obtained from the CCLE, including the reference MDA-MB-231 cell line in addition to our *SNAI1*-KOs and MDA-MB-231-WT cells, showed that *SNAI1*-KOs clustered away from WT and together with luminal (SUM52PE, SUM185PE, SUM44PE) and basal-B (HMC18, SUM159PT, SUM149PT) cells (Supplementary Fig. 2C, Supplementary Table 4). This finding made us examine more carefully the expression levels of some additional genes, which are established as markers of the basal and luminal BRCA classes [8–11]. The basal BRCA genes *KRT14* and *EGFR* were strongly downregulated in the *SNAI1*-KO cells (Supplementary Fig. 2D). In contrast, the presumed basal marker *SPARC* was undetectable in parental MDA-MB-231 cells and its expression was highly upregulated in the *SNAI1*-KO cells (Supplementary Fig. 2D). Conversely, the luminal marker *GRM4* that was weakly expressed in parental MDA-MB-231, was highly expressed in the *SNAI1*-KO cells, whereas *KRT8* and *AGR2* gave the profile of the basal markers (Supplementary Fig. 2D). This analysis suggests that the clustering analysis (Supplementary Fig. 2C, Supplementary Table 4) is reliable and gene-specific expression analysis can indicate the intermediate “space” of a partial phenotypic switch that occurred between basal and luminal gene profiles upon *SNAI1* depletion.

**Fig. 3:**
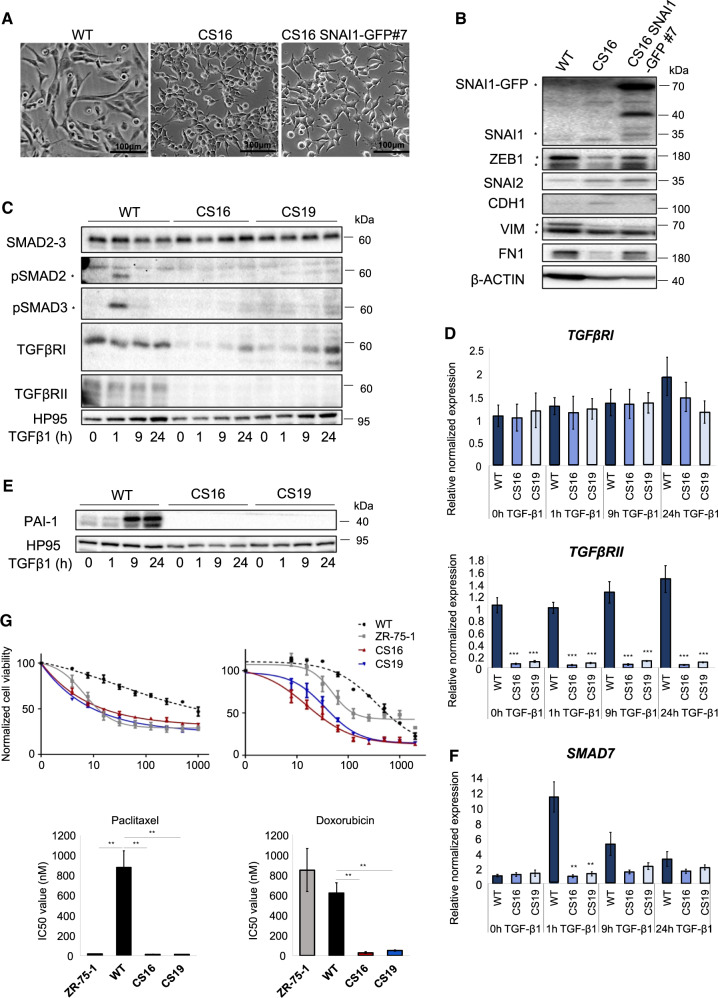
Loss of SNAI1 dysregulates TGFβ signaling and EMT pathways in MDA-MB-231 cells. **A** Representative phase contrast images from three biological repeats showing the morphology of MDA-MB-231-WT, CS16 and CS16 SNAI1-GFP#7 cells. Scale bars = 100 μm. **B** Protein expression levels of SNAI1 and of the EMT transcription factors ZEB1, SNAI2, of the epithelial protein CDH1 and of the mesenchymal proteins FN1, VIM in MDA-MB-231-WT, CS16 and CS16 SNAI1-GFP#7 cells. β-Actin serves as loading control. Stars indicate the specific protein bands; upper panel: stars correspond to SNAI1-GFP (top) and SNAI1 (bottom). **C** Protein expression levels of SMAD2/3, pSMAD2/3, TGFBRI and TGFBRII in MDA-MB-231-WT and *SNAI1*-KOs stimulated with 5 ng/ml of TGFβ1 for the indicated time periods. HP95 serves as loading control. Stars indicate the bands corresponding to pSMAD2 and pSMAD3. **D** RT-qPCR analysis of *TGFBRI*, *TGFBRII* mRNA levels in MDA-MB-231-WT and *SNAI1*-KO cells stimulated with 5 ng/ml of TGFβ1 for the indicated time periods. **E** Protein expression levels of PAI-1 in MDA-MB-231-WT and *SNAI1*-KOs stimulated with 5 ng/ml of TGFβ1 for the indicated time periods. HP95 serves as loading control. Immunoblots in **B**, **C** and **E** are representative of three biological replicates along with molecular mass markers in kDa. **F** RT-qPCR analysis of *SMAD7* mRNA levels in MDA-MB-231-WT and *SNAI1*-KOs stimulated with 5 ng/ml of TGFβ1 for the indicated time periods. Values in **D** and **F** represent relative mRNA expression normalized to *GAPDH*. Data in **D** and **F** are presented as mean values of three biological replicates ± SEM, each in technical triplicates and *p*-values are shown based on two-tailed unpaired Student’s *t*-test. **G** Cell viability assay of MDA-MB-231-WT, *SNAI1*-KOs and ZR-75-1 cells in increasing concentrations of paclitaxel and doxorubicin as measured by PrestoBlue after 48 hours. The data are shown as mean values of three biological replicates ± SEM, each in technical triplicates. *p*-values *p ≤ 0.05, **p ≤ 0.01, ***p ≤ 0.001.

To validate the transcriptomic data, we assessed cell phenotype and expression of epithelial and mesenchymal genes. *SNAI1*-KOs presented an altered and cobble-stoned morphology compared to the highly elongated WT cells (Fig. 2E). CDH1 and ZO-1 immunolocalization revealed fragmented but elevated expression in the plasma membrane, indicating the reappearance of rudimentary adherens and tight junctions between the *SNAI1*-KO cells (Fig. 2F). WT mesenchymal cells were characterized by complete absence of such junctions, while ZR-75-1 luminal BRCA cells were characterized by complete junctions (Fig. 2F). Furthermore, *SNAI1*-KO cells exhibited substantial networks of cortical F-actin assembly, characteristic of epithelial cells, in contrast to the stress fiber organization in WT cells (Fig. 2F). The partial loss of mesenchymal phenotype was confirmed by the decreased levels of the EMT-TF ZEB1 and of the mesenchymal proteins (FN1, VIM) and by increased levels of the epithelial CDH1 in *SNAI1*-KO cells, in the absence or presence of TGFβ1 (Fig. 2G, H). Stable SNAI1 overexpression in *SNAI1*-KO cells (CS16 SNAI1-GFP#7) reversed the partial epithelial phenotype (Fig. 3A, B). These results agree with the evidence that SNAI1 acts as a repressor of *CDH1* and tight junction genes [23–25, 55]. Thus, *SNAI1* knockout causes a phenotypic switch of basal mesenchymal cells towards an intermediate phenotype with acquired epithelial and retained mesenchymal characteristics.

In accordance with the RNA-sequencing data (Supplementary Fig. 2A), the expression of the TGFβ type I (TGFβRI, protein decrease without mRNA changes) and type II (TGFβRII, drastic decrease of both mRNA and protein) receptors and the phosphorylated form of their downstream mediators SMAD2/3 upon TGFβ1 stimulation, were decreased (Fig. 3C, D). The failure of TGFβ1 to stimulate its direct targets plasminogen activator inhibitor-1 (PAI-1) protein levels and inhibitory SMAD7 mRNA levels (Fig. 3E, F), further confirmed that *SNAI1* knockout dysregulated the TGFβ pathway. Rescuing *SNAI1* expression in the CS16 SNAI1-GFP#7 clone restored the levels of TGFβRII and the responsiveness of SMAD7 to TGFβ signaling (Supplementary Fig. 2E). TGFβ signaling has been linked to resistance to targeted and conventional anti-cancer agents via induction of EMT [56]. We thus investigated the cytotoxic response of *SNAI1*-KOs to paclitaxel and doxorubicin. As expected, *SNAI1*-KO cells were more sensitive to cytotoxic agents compared to WT cells (Fig. 3G). Thus, upon *SNAI1* knockout, TNBC-basal cells acquire a phenotype, distinct from their WT counterparts, which closely resembles a basal-luminal intermediate.

### *SNAI1* knockout diminishes invasion in culture and in vivo

**Fig. 4:**
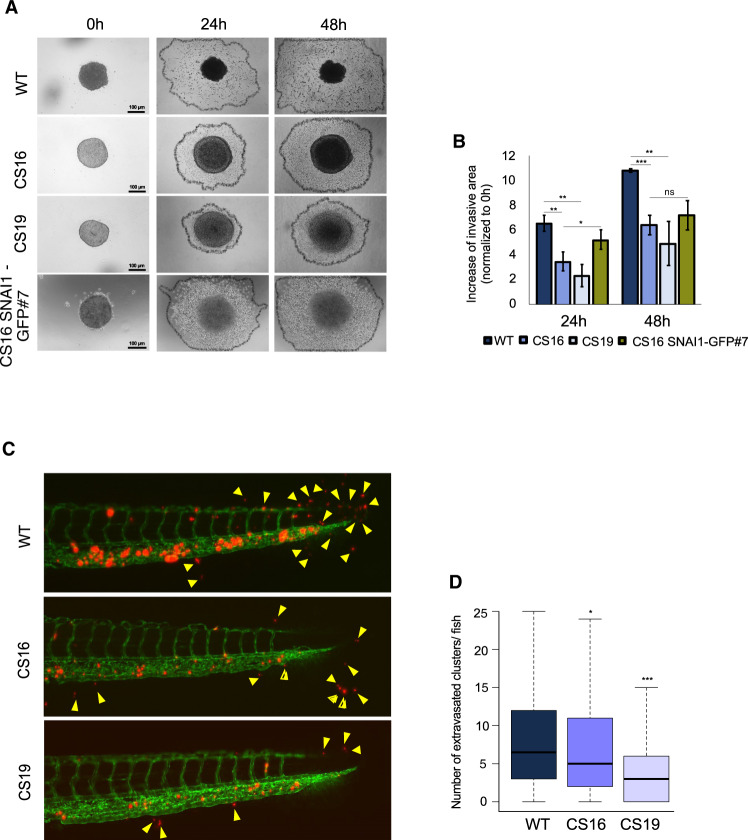
*SNAI1* knockout reduces cell invasion. **A**, **B** Representative phase-contrast images from three biological repeats of MDA-MB-231-WT, *SNAI1*-KOs, ZR-75-1 and CS16 SNAI1-GFP#7 mammospheres embedded in collagen. Black lines demarcate the outer rim formed by invasive cells after 24 or 48 h (**A**) with quantification of the invasion area subtracted by the mammosphere area (**B**). Data are presented as mean values ± SEM of three biological replicates, each in technical triplicates and *p*-values are shown based on two-tailed unpaired Student’s *t*-test. **C**, **D** Representative images of zebrafish 24 h post-implantation with GFP-positive vasculature and CM-Dil dye-positive red cells. Extravasated cell clusters are indicated with yellow arrows (**C**). Quantification of extravasated cell clusters and associated significance (**D**). Data values from 150 WT, 142 CS16 and 152 CS19 zebrafish per cell line are presented as mean values ± SEM and *p*-values are shown based on two-sided Wilcoxon sum-rank *U* test. *p*-values *p ≤ 0.05, **p ≤ 0.01, ***p ≤ 0.001.

We next assessed the invasive ability of MDA-MB-231-WT and *SNAI1*-KO cells by embedding 3D mammospheres in a collagen matrix and observing invasion. While WT cells massively invaded the surrounding matrix, *SNAI1*-KOs presented a reduced invasive ability, which was rescued after stable SNAI1 overexpression (Fig. 4A, B). Invasiveness in vivo was assessed by injecting CM-Dil Dye-labeled MDA-MB-231-WT and *SNAI1*-KO cells in the duct of Cuvier of transgenic Tg (Fli1:EGFP) zebrafish embryos. A higher number of WT cell clusters extravasated from the blood vessels and invaded the collagenous matrix of the tail when compared to *SNAI1*-KO cells (Fig. 4C, D). In conclusion, *SNAI1* knockout decreased the invasive potential of TNBC-basal MDA-MB-231 cells.

### The *SNAI1* knockout cell population is highly proliferative with high mammosphere-forming capacity and exhibits an altered stem cell repertoire

**Fig. 5:**
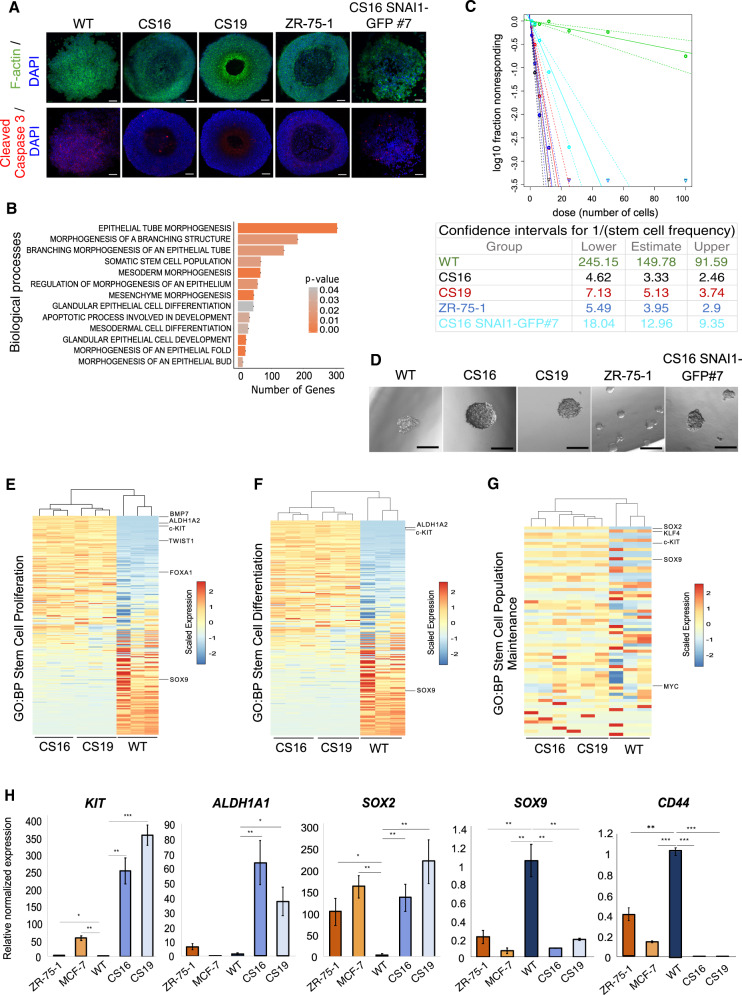
*SNAI1* knockout drives the generation of a population enriched in epithelial stem-like/progenitor cells with increased mammosphere-forming ability. **A** Representative immunofluorescence staining pictures from three independent biological repeats of F-actin (green) and cleaved caspase 3 (red) in mammospheres derived from MDA-MB-231-WT, *SNAI1*-KOs, ZR-75-1 and CS16 SNAI1-GFP#7 cells. Nuclei are stained in blue with DAPI. Scale bars = 50 μm. **B** Bar graph showing the biological processes relevant to breast morphogenesis enriched in *SNAI1*-KOs. The horizontal axis indicates the number of genes annotated to each process and the color code represents the corresponding *p*-value. **C** ELDA depicting the stem cell frequency of MDA-MB-231-WT (green curve), *SNAI1*-KO CS16 (black curve), CS19 (red curve), ZR-75-1 (blue curve) and CS16 SNAI1-GFP#7 (turquoise curve) cells cultured for 10 days. The number of wells containing spheres for each plating density is plotted. Steeper slopes indicate higher frequencies of sphere-forming cells. The table indicates the average stem cell frequency per cell group of three independent biological replicates. **D** Representative phase contrast images from three biological repeats showing MDA-MB-231-WT, *SNAI1*-KO, ZR-75-1 and CS16 SNAI1-GFP#7 mammospheres from the experiment in **C**. Scale bars = 100 μm. **E**–**G** Heatmaps demonstrating expression levels of differentially expressed genes involved in stem cell-related biological processes based on gene set enrichment analysis. The color-coded scales represent normalized counts per million values of WT and *SNAI1*-KO cells. **H** RT-qPCR analysis of *KIT*, *ALDH1A1*, *SOX2*, *SOX9* and *CD44* mRNA levels in ZR-75-1, MCF-7, MDA-MB-231-WT, and *SNAI1*-KO cells. Values represent fold-change of mRNA expression normalized to *GAPDH* and expressed relative to the level in MDA-MB-231 cells. Data are presented as mean values of three biological replicates ± SEM, each in technical triplicates and *p*-values are shown based on two-tailed unpaired Student’s *t*-test. *p*-values *p ≤ 0.05, **p ≤ 0.01, ***p ≤ 0.001.

*SNAI1*-KOs presented an enhanced ability to form mammospheres in 3D culture (Figs. 4A, 5A, Supplementary Fig. 3A). MDA-MB-231-WT mammospheres formed multilayered solid structures without a defined outer layer edge (Figs. 4A, 5A, Supplementary Fig. 3A). On the contrary, *SNAI1*-KOs, formed mammospheres showing differentiated morphology of an outer layer of cells with defined edges around a lumen; the lumen was generated via cell death, as indicated by an increased number of cleaved caspase 3-positive cells in the center of the spheres (Figs. 4A, 5A, Supplementary Fig. 3A). This phenotype represents differentiated acini. Rescuing *SNAI1* expression restored the multilayered, lumen-less mammosphere phenotype and suppressed the cleaved caspase 3-positive cells that form the lumen (Fig. 5A). *SNAI1*-KO mammosphere morphology resembled that of ZR-75-1 cells (Figs. 4A, 5A, Supplementary Fig. 3A). Interestingly, *SNAI1*-KO and ZR-75-1 mammospheres exhibited a larger cross-section area compared to WT mammospheres (Supplementary Fig. 3B). The latter could be due to the increased proliferation of *SNAI1*-KOs compared to WT, but not of ZR-75-1 cells (Supplementary Fig. 3C). In accordance with our observations, pathway-enrichment analysis of the common genes in *SNAI1*-KOs revealed that biological processes relevant to breast morphogenesis were significantly upregulated, including epithelial tube morphogenesis and apoptotic process involved in development (Fig. 5B, Supplementary Table 3).

To assess further the advanced competence of *SNAI1*-KOs to form mammospheres, we tested their self-renewal capacity, which was approximately 37-fold higher than WT cells, based on ELDA measurements (Fig. 5C). Interestingly, *SNAI1*-KO cells resembled ZR-75-1 cells in terms of stem cell frequency, even though the KO cells tended to generate bigger spheres originating from the same original cell number (Fig. 5C, D). Rescuing *SNAI1* expression reduced the self-renewal frequency by 3- to 4-fold, which had a clear tendency to reach the parental cell phenotype (Fig. 5C). Similarly, gene set enrichment analysis demonstrated that stem cell-associated biological processes were significantly enriched in *SNAI1*-KO cells (Fig. 5E–G). Among other genes, we validated the expression of *ALDH1A1*, *KIT*, *SOX2*, *SOX9* and *CD44* (Fig. 5H). Of note, KIT^+^, ALDH1^+^ and SOX2^+^ cancer stem cell populations harbor proliferative and epithelial-like characteristics, while CD44^+^ and SOX9^+^ cancer stem cell populations demonstrate invasive and mesenchymal-like properties [15, 57, 58]. Supporting this notion, this validation of the RNA-sequencing data showed a notable increase in *KIT*, *ALDH1A1* and *SOX2* mRNA levels in *SNAI1*-KO cells compared to WT cells, mimicking but significantly exceeding the expression of these genes in the luminal cell lines ZR-75-1 and MCF-7 (Fig. 5H, Supplementary Fig. 3D). On the contrary, a significant reduction in the mRNA expression levels of *CD44* and *SOX9* was observed in *SNAI1*-KO cells, expressing a molecular phenotype more similar to the luminal cells (Fig. 5H, Supplementary Fig. 3D). Stable SNAI1 overexpression in CS16 *SNAI1*-KO cells (*SNAI1*-GFP#7) reversed the expression of *KIT* and *ALDH1A1* but not of *CD44* (Supplementary Fig. 3E).

Remarkably, the similarities in the expression patterns of the tested genes, hierarchically clustered *SNAI1*-KO cells closer to the luminal cells than their WT counterparts (Supplementary Fig. 3D). This observation suggests that the stem cell-associated gene profiles reflect the differentiation state and growth features of the mammospheres (Fig. 5A, Supplementary Fig. 3A). Thus, the *SNAI1*-KO cells have clearly acquired more epithelial-like features while they maintain reduced levels of their mesenchymal state. Collectively, the data suggest that loss of *SNAI1* in TNBC-basal cells provides plasticity of developmental potential consistent with a switch towards a mammary stem/progenitor cell phenotype, which gives rise to more differentiated mammospheres. Although stem-like features are retained by both WT and *SNAI1*-KO cells, the more epithelial *SNAI1*-KO cells clearly gain stemness potential in 3D cultures, characterized by KIT and ALDH1A1 overexpression.

### Differentiation plasticity of *SNAI1* knockout cells engages FOXA1

**Fig. 6:**
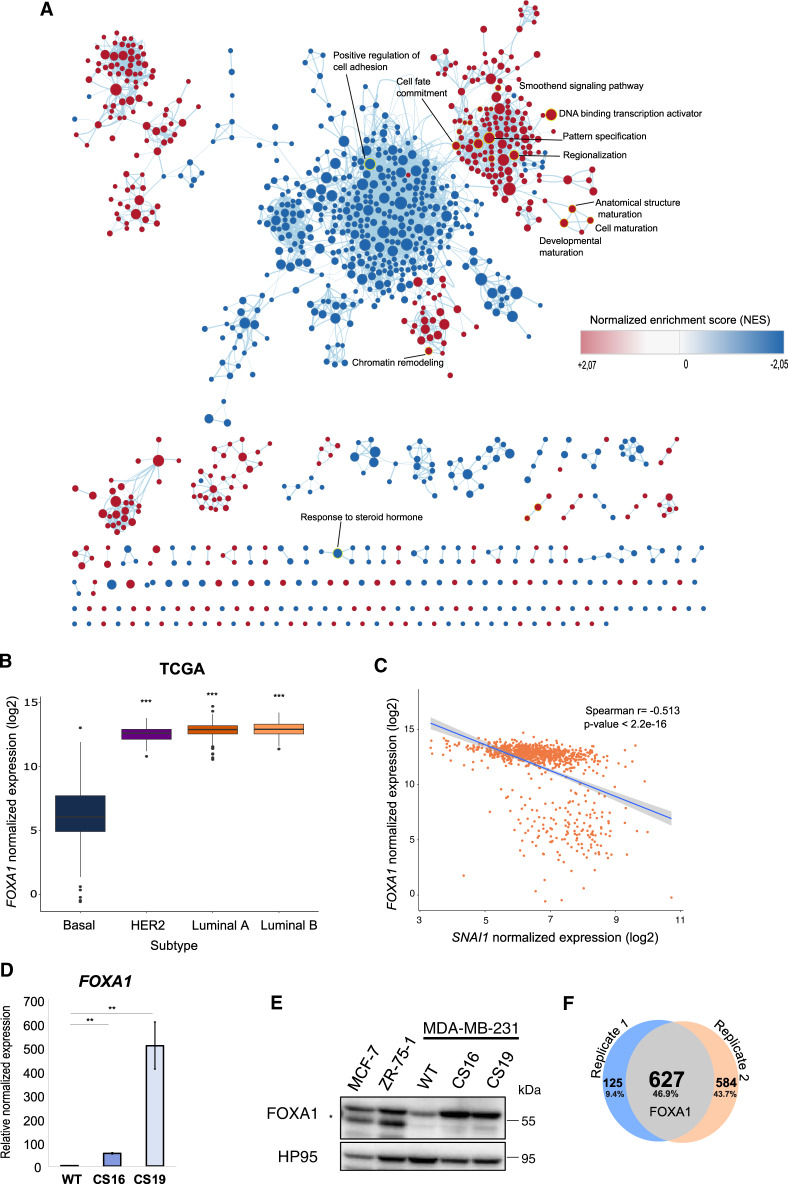
FOXA1 is involved in the differentiation plasticity of *SNAI1* knockout cells and its expression is regulated by SNAI1. **A** Network analysis visualizing the interactions between significantly enriched biological processes in *SNAI1*-KOs (*p*-value < 0.005 and overlap coefficient ≥ 0.5). Each node represents one biological process, and the size of the node corresponds to the number of the constituting genes. The lines refer to the significantly connected processes. FOXA1 is involved in the highlighted processes. The color code indicates the normalized enrichment score. **B** Expression of *FOXA1* in various human BRCA subtypes obtained from TCGA datasets (Basal *n* = 171, HER2 *n* = 78, Luminal A *n* = 499, Luminal B *n* = 197). The statistical significance is derived using the Wilcoxon rank-sum test (*U* test). **C** Scatter plot demonstrating the negative correlation between *SNAI1* and *FOXA1* expression in breast cancer samples obtained from TCGA datasets. **D** RT-qPCR analysis of *FOXA1* in MDA-MB-231-WT and *SNAI1*-KOs. Values represent fold-change of mRNA expression normalized to *GAPDH*. Data are presented as mean values of three biological replicates ± SEM, each in technical triplicates and *p*-values are shown based on two-tailed unpaired Student’s *t*-test. **E** Representative immunoblot of three biological replicates along with molecular mass markers in kDa showing protein expression levels of FOXA1 in MCF-7, ZR-75-1, MDA-MB-231-WT and *SNAI1*-KOs. HP95 serves as loading control. A star indicates the specific protein band detected by the FOXA1 antibody. **F** Venn diagram showing the numbers of the significantly enriched annotated peaks obtained from SNAI1 ChIP-sequencing analysis in human colorectal cancer cells. *p*-values *p ≤ 0.05, **p ≤ 0.01, ***p ≤ 0.001.

To address the mechanism of the observed plasticity of the *SNAI1*-KO cells, we investigated the transcriptomic data and found the estrogen signaling mediator *FOXA1* [59] among the highly upregulated genes in *SNAI1*-KOs (Fig. 2A). FOXA1 expression associates with the luminal tumor subtypes, as defined by ER and/or PGR positivity and HER2 negativity [60, 61]. Network analysis visualizing the interactions between significantly enriched biological processes in *SNAI1*-KO cells revealed that *FOXA1* is involved in numerous processes related to chromatin remodeling, cell fate commitment and developmental maturation (Fig. 6A). Analyzing TCGA datasets across BRCA subtypes showed that *FOXA1* expression levels were significantly lower in TNBC-basal tumors, highly expressing *SNAI1*, compared to the other molecular subtypes (Fig. 6B). Accordingly, basal-B BRCA cell lines expressed significantly lower levels of *FOXA1* compared to HER2 and luminal BRCA cells provided by the CCLE (Supplementary Fig. 4A, B). Correlation analyses across the different molecular subtypes of BRCA in TCGA further supported a negative association between *FOXA1* and *SNAI1* expression (Fig. 6C, Supplementary Fig. 4C). In line with this, mRNA and protein analyses revealed a notable increase in the levels of FOXA1 in *SNAI1*-KO cells compared to WT, while ZR-75-1 and MCF-7 luminal cells presented the highest expression (Fig. 6D, E). Stable SNAI1 overexpression in CS16 *SNAI1*-KO (*SNAI1*-GFP#7) cells reversed, but only slightly, the expression of FOXA1 at both mRNA and protein levels (Supplementary Fig. 4D, E). This might be explained by the presence of a strong positive inducer of *FOXA1* expression that does not respond to the presence of ectopic SNAI1. Interestingly, re-annotation of the significantly enriched peaks obtained from SNAI1 ChIP-sequencing analysis in human colorectal cancer cells [27] showed that SNAI1 occupies the proximal promoter region of *FOXA1* (Fig. 6F, Supplementary Table 5). This observation consolidates our data concerning the SNAI1-mediated repression of *FOXA1* in BRCA cells. In addition, regression analysis of *FOXA1* and *SNAI1* expression in TCGA BRCA samples demonstrated that only 15% of *SNAI1* expression is affected by *FOXA1* as indicated by the adjusted R-square value (Supplementary Fig. 4F). This suggests that SNAI1 acts upstream of FOXA1 and not vice versa. Overall, these findings demonstrate that SNAI1 represses FOXA1 expression, whereas *FOXA1* transcriptional induction has a potential functional role in the plasticity of TNBC-basal cells in terms of differentiation caused upon *SNAI1* knockout.

### *SNAI1* knockout drives differentiation towards androgen-responsive breast cancer cells through FOXA1

**Fig. 7:**
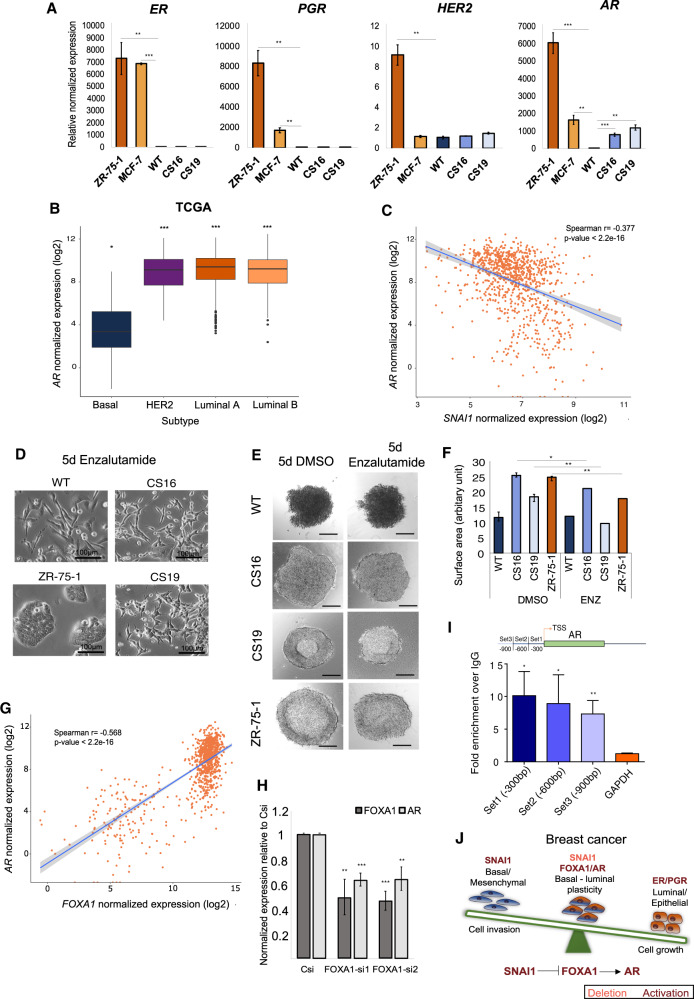
FOXA1 associates with the differentiation plasticity of *SNAI1* knockout cells via regulation of AR expression. **A** RT-qPCR analysis of *ER*, *PGR*, *HER2* and *AR* in ZR-75-1, MCF-7, MDA-MB-231-WT and *SNAI1*-KO cells. **B** Boxplots showing the expression of *AR* in various human BRCA subtypes in TCGA datasets (Basal *n* = 171, HER2 *n* = 78, Luminal A *n* = 499, Luminal B *n* = 197). The statistical significance is derived using the Wilcoxon rank-sum test (*U* test). **C** Scatter plot demonstrating the negative correlation between *SNAI1* and *AR* expression in BRCA samples obtained from TCGA datasets. **D** Representative phase contrast images from three biological repeats showing the morphology of MDA-MB-231-WT, *SNAI1*-KOs and ZR-75-1 cells at day 5 of treatment with 0.05 μM of the androgen antagonist enzalutamide. Scale bars = 100 μM. **E** Representative phase contrast images from three biological repeats showing the morphology of mammospheres derived from MDA-MB-231-WT, *SNAI1*-KOs and ZR-75-1 cells at day 5 of treatment with either DMSO or 0.05 μM enzalutamide. Scale bars = 200 μM. **F** Quantification of the cross-section area of the mammospheres. **G** Scatter plot demonstrating the positive correlation between *FOXA1* and *AR* expression in BRCA samples obtained from TCGA datasets. **H** RT-qPCR analysis of *FOXA1* and *AR* expression in CS16 *SNAI1*-KO after knocking down FOXA1 expression using two independent siRNAs. Values in **A** and **H** represent fold-change of mRNA expression normalized to *GAPDH*. Data in **A**, **F** and **H** are presented as mean values of three biological replicates ± SEM, each in technical triplicates and *p*-values are shown based on two-tailed unpaired Student’s *t*-test. **I** ChIP-qPCR analysis of FOXA1 binding in three different regions (900, 600 and 300 bp) upstream of the *AR* transcription starting site. Values represent fold-enrichment over the IgG and FOXA1 binding to *GAPDH* is used as a negative control. Data are presented as mean values of three biological replicates ± SEM, each in technical triplicates and *p*-values are shown based on one-tailed unpaired Student’s *t*-test. **J** Schematic illustration of the proposed model of cellular plasticity in breast cancer based on the SNAI1/FOXA/AR regulatory module. Dark letters: high expression; light letters: low expression. *p*-values *p ≤ 0.05, **p ≤ 0.01, ***p ≤ 0.001.

As reported in earlier studies, *FOXA1* expression correlates with the luminal subtypes and directly binds to the promoter of *ER* to activate its expression [59–61]. This, in combination with a significant alteration in the expression of biological processes related to steroid hormone biosynthesis and metabolism observed in *SNAI1*-KO cells (Supplementary Fig. 5A, Supplementary Table 3), prompted us to investigate further the expression levels of steroid hormone receptors linked to the luminal and HER2 BRCA subtypes. Remarkably, *SNAI1*-KOs did not alter mRNA levels of *ER, PGR* or *HER2*, but exhibited dramatically enhanced *androgen receptor* (*AR*) mRNA levels compared to WT cells (Fig. 7A). The *AR* can be expressed in both ER^+^ and ER^−^ BRCA and its role in driving breast cancer growth depends on ER co-expression [62]. Stable SNAI1 overexpression in CS16 *SNAI1*-KO (*SNAI1*-GFP#7) cells reduced *AR* mRNA expression (Supplementary Fig. 5B). Using TCGA datasets, we found that TNBC-basal tumors harbor lower *AR* levels compared to the other molecular subtypes (Fig. 7B). In agreement with this, the basal-B BRCA cell lines expressed significantly lower levels of *AR* compared to the HER2 and luminal BRCA cells provided by the CCLE (Supplementary Fig. 5C, D). Importantly, *SNAI1* negatively correlated with *AR* expression across BRCA molecular subtypes (Fig. 7C), which is mostly attributed to the luminal A and B subtypes (Supplementary Fig. 5E).

To further assess the role of AR in the observed plasticity of cell differentiation, we treated *SNAI1*-KO and ZR-75-1 cells with low concentrations of the AR antagonist, enzalutamide. We observed that cells acquired an elongated morphology, which was more pronounced in the *SNAI1*-KOs (Fig. 7D), most probably due to the lower expression of AR and significant expression of EMT-TFs (Fig. 2G, H). The proliferative and invasive capacities of the cells remained unaffected by enzalutamide treatment (Supplementary Fig. 5F, G). Interestingly, upon enzalutamide treatment, we observed deformation of the lumen in the differentiating spheres, which was more pronounced in *SNAI1*-KO and ZR-75-1 mammospheres, while the cross-section area of all spheres was significantly decreased (Fig. 7E, F). It should be noted that enzalutamide acting on parental MDA-MB-231 cells did not affect their spindle-like morphology (Fig. 7D), the aggregated, multilayered mammosphere phenotype (Fig. 7E) or the mammosphere surface area (Fig. 7F). Even though further work is required to define the exact mechanism of action of AR in the absence of SNAI1 expression, these data suggest that AR signaling is important for the basal-luminal plasticity observed in *SNAI1*-KO cells and that its blockade tends to transit the cells back to their basal phenotype even in the absence of *SNAI1*.

In TNBC-basal subtypes, when co-expressed with *FOXA1*, *AR* mediates an estrogen-like expression signature, similar to luminal BRCA [63]. Accordingly, we found a positive correlation between *FOXA1* and *AR* expression in breast tumors (Fig. 7G, Supplementary Fig. 5H), while transient silencing of *FOXA1* led to decreased *AR* mRNA expression levels in *SNAI1*-KO cells (Fig. 7H). In addition, regression analysis of *FOXA1* and *AR* expression in TCGA BRCA samples demonstrated that *FOXA1* could predict the variation in *AR* expression as 63% being affected by *FOXA1* (Supplementary Fig. 5I). We next asked whether FOXA1 binds directly to *AR* gene sequences, promoting *AR* expression in breast cancer cells. To this end, we re-annotated the genomic locations of the FOXA1 peaks provided by ChIP-sequencing data performed in human MCF-7 BRCA cells [64] and found a significant enrichment of FOXA1 binding to the *AR* promoter upon FOXA1 overexpression (+Dox) which was ceased in the absence of FOXA1 induction (−Dox) (Supplementary Fig. 5J, Supplementary Table 6). Remarkably, we observed that FOXA1 occupies its own promoter, suggesting a feedforward loop of FOXA1 expression regulation (Supplementary Fig. 5K). However, we did not find noticeable global enrichment of androgen receptor element (ARE) sequences across the identified peaks of FOXA1 binding to the genome (Supplementary Fig. 5K). We therefore performed ChIP-qPCR analysis in CS16 *SNAI1*-KO cells, in order to validate FOXA1 binding to the *AR* promoter, and we could observe a significant enrichment of FOXA1 binding (over the IgG control) using three different sets of primers binding to −900, −600 and −300 bp upstream of the *AR* transcriptional start site (Fig. 7I). Collectively, these data demonstrate that upon *SNAI1* knockout, FOXA1 possibly induces its own expression and further induces AR expression, which in turn provides basal-luminal plasticity to the cells (Fig. 7J).

### FOXA1 distinguishes between basal and non-basal breast cancer subtypes

Our results demonstrate that upon *SNAI1* knockout, MDA-MB-231 cells acquire a plastic phenotype between basal and luminal BRCA cells due to FOXA1 and AR upregulation. So, we sought to explore whether a combination of SNAI1/FOXA1/AR expression could classify the basal/luminal intermediate state. To this end, we utilized a mathematical formula to describe an index as [(log_2_(1+*FOXA1* expression) + (log_2_(1+*AR* expression)]/(log_2_(1+*SNAI1* expression) in TCGA BRCA datasets. Interestingly, we found that the median value of the index differs significantly among BRCA subtypes, with the basal subtype presenting the lowest and luminal B the highest values (Supplementary Fig. 6A). Thus, utilizing the expression index in addition to *SNAI1*, *FOXA1* and *AR* expression levels, we performed a multinomial logistic regression analysis to build a model that could predict the classification of BRCA molecular subtypes based on these three genes. Surprisingly, the model indicated that *FOXA1* is the only significant variable (Supplementary Fig. 6B). However, after constructing a confusion matrix to calculate the accuracy of the model in predicting the correct subtype, we found that the model predicts the basal subtype with 97% accuracy and the luminal A subtype with 99% accuracy (Supplementary Fig. 6B). Nevertheless, the model neither predicts the HER2 subtype nor discriminates between luminal A and luminal B subtypes. To further test the validity of this transcription factor signature, we performed a binomial logistic regression analysis utilizing the *FOXA1* expression as a variable and dividing TCGA BRCA tumors into basal and non-basal. This classification incorporated HER2, luminal A and luminal B into a single, non-basal category. *FOXA1* expression could significantly discriminate between basal and non-basal tumors with higher performance measured by the receiving operating character (ROC) curve analysis (Supplementary Fig. 6C, D). Collectively, our data indicate that *FOXA1* expression is essential in promoting cellular plasticity across the basal/luminal spectrum which is mainly dictated by the EMT-TF SNAI1 in breast cancer cells.

## Discussion

Understanding lineage plasticity, the ability of differentiated/committed cells to transit to a more progenitor-like stage that can then generate another cell type, during breast cancer initiation and development, is crucial in formulating preventive and therapeutic strategies. SNAI1, a well-established inducer of EMT during embryonic development, is also active in a variety of adult epithelial cells, where it drives the differentiation of mesenchymal stem cells and cancer-associated fibroblasts, fueling tumors with aggressive, metastatic and chemoresistant properties [21, 22, 53, 65, 66]. In the current study, we queried the function of SNAI1 in regulating lineage plasticity during breast cancer development and three major key findings have been accomplished after CRISPR/Cas9-mediated knockout of *SNAI1* in the MDA-MB-231 TNBC-basal cells: (a) The mutant cells reside within a separate breast cancer differentiation cluster than their WT counterparts based on their RNA expression profiles (Supplementary Fig. 2C, Supplementary Table 4) and have acquired an epithelio-mesenchymal phenotype, which associates with a parallel decrease in TGFβ signaling, cell invasion and drug resistance (Figs. 2–4, Supplementary Fig. 2). (b) Although ectopic SNAI1 overexpression usually associates with stem-like features among mesenchymal breast cancer cells [12], its loss generates quasi-epithelial cells with the stem/progenitor phenotype, which causes retention of the stem-like features of the cells, based on preferential expression of a new set of stemness proteins (Fig. 5, Supplementary Fig. 3). (c) The mutant cells transit to a phenotype that allows the expression of the transcription factors FOXA1 and AR (Figs. 6, 7, Supplementary Figs. 4, 5). FOXA1 suppresses the basal phenotype of breast cancer cells [67] (Supplementary Figs. 5, 6) and is required for AR binding to target genes promoting breast cancer cell growth and proliferation [68].

Transcriptomic analysis revealed the existence of approximately 6000 differentially expressed genes between *SNAI1*-KOs and WT cells, further resulting in the different hierarchical clustering of the *SNAI1*-KO cells along 61 BRCA cell lines (Fig. 2A, B, D, Supplementary Fig. 2C). Analysis of established proteins of the epithelial and mesenchymal program of breast cancer cells clearly supports the notion that loss of SNAI1 enforces an epithelio-mesenchymal phenotype (Fig. 2E–H). This evidence also agrees with previous reports which concluded that SNAI1 regulates the expression of several genes associated with mesenchymal cell differentiation [21, 28, 30, 66]. Architecturally, CDH1 and ZO-1 generated rudimentary cell adherens and tight junctions, respectively, with specific areas of focal assembly (more pronounced for ZO-1) (Fig. 2F), pointing to a remaining semi-dominant, compensatory action of other EMT-TFs, e.g. ZEB1 in the *SNAI1*-KO cells (Fig. 2G, H). Although no evidence of a disturbance of the expression profile of *SNAI2* could be obtained based on RNA-sequencing (Supplementary Fig. 1F), protein analysis revealed higher levels of SNAI2 protein in the CS16 *SNAI1*-KO clone (Fig. 2G, H). This observation could be interpreted as a mechanism of compensation of SNAI2 for the loss of SNAI1. Yet, this result was not reproducible in CS19 cells, neither was SNAI2 protein level reverted in the rescue clone CS16 *SNAI1*-GFP#7 (Fig. 3B). We therefore hypothesize that a post-translational, stabilizing mechanism enhanced the levels of SNAI2 in CS16, probably independent from the action of SNAI1, which is known to transcriptionally induce SNAI2. Furthermore, the elevated SNAI2 protein did not provide functional output as measured by the reduced migration, invasion and extravasation of the *SNAI1*-KO cells (Fig. 4), or the loss of responses to TGFβ signaling (Fig. 3). We presume aberrant expression of SNAI2 protein in C16 cells, without evidence for biological activity and thus, we cannot propose a firm compensatory action of SNAI2 in the presence of *SNAI1* loss. In addition, the downregulation of TGFβ signaling upon *SNAI1* knockout in MDA-MB-231 cells and the inferred reduction of drug resistance and cell invasiveness (Figs. 3, 4), are well in accordance with reports indicating the implication of TGFβ signaling in the upregulation of EMT and collaboration of downstream targets of TGFβ with SNAI1 in order to repress the expression of epithelial genes, including *CDH1*, *CAR* and *occludins* [69, 70]. In agreement with our model of TNBC-basal cell reprogramming towards the luminal lineage upon *SNAI1*-KO, SNAI1 overexpression in luminal BRCA cells was shown to repress *ER* and to upregulate TGFβ signaling, resulting in cell junction destruction and acquisition of highly mesenchymal and invasive phenotypes [71].

The most notable observation of the present study emanated from the detailed morphological inspection of 3D mammospheres generated by *SNAI1*-KO cells and their comparison to luminal ZR-57-1 cells (Figs. 4A, 5A, Supplementary Fig. 3A). The resemblance of mammosphere architecture accompanied with transcriptomic enrichment of biological processes relevant to normal mammary gland morphogenesis (Fig. 5B), suggested differentiation similarities between luminal and basal BRCA cells that lack *SNAI1* expression. Interestingly, *SNAI1*-KO cells presented higher stem cell frequency and underwent a switch in the expression of cancer stemness signaling genes, with significant upregulation of epithelial *KIT*, *ALDH1A1* and *SOX2*, and analogous downregulation of mesenchymal *CD44* and *SOX9* (Fig. 5C–H). This endowed cells with increased proliferative and reduced invasive potential and the capacity to form well-differentiated mammospheres, as others have similarly reported for BRCA stem cells of the luminal lineage [15, 57, 58, 72]. The switch between the expression of the aforementioned stemness genes allows breast cancer cells to exhibit plasticity as a common mechanism of generating competent transitory cells during oncogenesis. This notion is further supported by studies trying to unravel the origin of breast cancer cells and tumor heterogeneity [1]. For example, the majority of basal-like tumors are derived from luminal progenitors where SOX9 drives luminal to basal plasticity during basal-like breast cancer development [11, 73]. In a similar concept, we provide evidence that *SNAI1-*deficient cells cluster together with luminal cells based on the expression of certain stem/progenitor-associated genes (Supplementary Fig. 3D).

The differentiation reprogramming of TNBC-basal cells carrying *SNAI1* knockout correlated with FOXA1 upregulation (Fig. 6, Supplementary Fig. 4) and the lower expression levels of *FOXA1* in basal tumors coupled with its negative correlation with *SNAI1* (Fig. 6B, C, Supplementary Fig. 4A–E), agree with the proposed tumor suppression function of FOXA1 in breast cancer cells [67]. A direct link between SNAI1 and FOXA1 is thus proposed based on reports from colorectal cancer where SNAI1 can directly repress the *FOXA1* gene, and tumor cells acquire mesenchymal features upon *FOXA1* knockout [27, 74]. In a similar scenario, the EMT-TF TWIST1 binds to the promoter and represses *FOXA1* in basal BRCA cells [75]. FOXA1 binds and opens highly compacted chromatin regions, allowing other TFs, including steroid hormone receptors, to initiate transcriptional programs [76]. In the mammary gland, FOXA1 acts as a pioneer factor for *ESR1* transcription and defines estrogen-dependent mammary epithelial differentiation [59, 77, 78]. However, we could not verify upregulation of the expression of neither *ER* nor *PGR* in the TNBC-basal *SNAI1*-KO cells (Fig. 7A). On the contrary, we identified a remarkable increase of *AR* expression which rendered the cells responsive to androgens, promoting epithelial differentiation under 2D and 3D conditions (Fig. 7D–F). In addition to regulating *ESR1* gene expression, FOXA1 acts as a pioneer factor for *AR* expression not only in prostate [79] but also in breast cancer, and, TNBCs that lose metastatic potential co-express *FOXA1* and *AR* [63, 68]. Our data agree with these previous reports and show a clear positive correlation between *FOXA1* and *AR* expression in BRCA (Fig. 7G, H, Supplementary Fig. 5H, I). This model is further strengthened by ChIP analysis, verifying the binding of FOXA1 to the *AR* gene regulatory sequences in the *SNAI1*-KO model (Fig. 7I). Interestingly, AR binds and regulates *ESR1 cis*-regulatory elements and its expression in patients with triple-negative carcinomas is associated with the luminal-AR subgroup that presents favorable outcome in terms of disease-free and overall survival [80–82]. Thus, we hypothesize that FOXA1-driven AR expression in TNBC-basal *SNAI1*-mutant cells triggers a transcriptional program reminiscent of ER-mediated transcription in luminal breast cancer, whose exact mechanism awaits further investigation.

In summary, we propose that upon *SNAI1* knockout, TNBC-basal cells are reprogrammed to the hybrid epithelio-mesenchymal phenotype of the EMT spectrum, while further generating basal-luminal plasticity as indicated by re-expression of FOXA1 and AR, and by shifting the differentiation balance towards a more proliferative and less invasive breast cancer cell population (Fig. 7J, Supplementary Fig. 7).

## Supplementary information

Supplementary data

Reporting summary

Dataset 1

Dataset 2

Dataset 3

Dataset 4

Dataset 5

Dataset 6

## Data Availability

All primary RNA-sequencing data have been deposited to GEO (accession number GSE210870; https://www.ncbi.nlm.nih.gov/geo/query/acc.cgi?acc=GSE210870). Processed RNA-sequencing data and database analysis files are presented in Tables S1–S6; further details are available upon reasonable request.
