# Supplementary material for: Loss of SNAI1 induces cellular plasticity in invasive triple-negative breast cancer cells
Source: Cell Death Dis. 2022 Sep 28;13(9):832. doi: 10.1038/s41419-022-05280-z (PMC9519755; doi:10.1038/s41419-022-05280-z)
Supplement: Supplementary file 1 — Supplementary data [file 41419_2022_5280_MOESM1_ESM.pdf]

Loss of SNAI1 induces cellular plasticity in invasive triple-negative breast cancer cells

Chrysoula Tsirigoti, Mohamad Moustafa Ali, Varun Maturi, Carl-Henrik Heldin and Aristidis Moustakas

SUPPLEMENTARY INFORMATION

SUPPLEMENTARY TABLES

Supplementary Table 1: Annotated_filtered_FDR0.05_FC2_SNAI_KO_vs_WT. Excel file listing the 7 824 differentially expressed genes in the *SNAI1*-KO cells relative to the parental WT MDA-MB-231 cells (3 437 upregulated genes and 4 387 downregulated genes) after filtering gene expression based on log2 fold-change ±2 and false discovery rate (FDR) < 0.05.

Supplementary Table 2: DEGs_Filtered_FDR0.05_FC2_Annotated_CS16_CS19. Excel file listing the 5 957 common differentially expressed genes between the two *SNAI1*-KO clones (CS16 and CS19). The gene list was derived from Table S1 by selecting the common DEGs. The table contains three sheets, one per knockout clone and the third is the common gene list.

Supplementary Table 3: SNAI_KO_vs_WT_DEGs_FC2_BP_KEGG_REAC_TF. Excel file listing all the gene ontology terms represented in 5 957 genes of Table S2 and analyzed based on the following categories: biological process (BP), KEGG database terms, Reactome database terms (REACT) and transcription factor terms (TF).

Supplementary Table 4: CCLE_Merged_log_TPM_Cell_lines+SNAI. Excel file listing expression values of 18 817 genes from the CCLE across 61 breast cancer cell lines, including the 9 samples of the MDA-MB-231-WT and *SNAI1*-KO cells from this experiment.

Supplementary Table 5: SNAI_ChIP-Seq_annotated_peaks-replicate-1_2. Excel file listing the re-annotation of the significantly enriched peaks obtained from the SNAI1 ChIP-sequencing analysis in human colorectal cancer cells and used to populate the data of Fig. 6F. The file contains three sheets, one for each of the two replicates stemming from the ChIP-sequencing experiment and the third listing the overlap of the annotated peaks.

Supplementary Table 6: FOXA1_ChIP-Seq+DOX_-DOX_annotated. Excel file listing the re-annotation of the genomic locations of the FOXA1 peaks provided by ChIP-sequencing data performed in human MCF-7 breast cancer cells upon FOXA1 overexpression (+Dox) or not (-Dox) and used to populate the data of Supplementary Fig. 5K. The file contains three sheets, annotation of the -Dox, the +Dox and the overlap used for the final analysis.

Supplementary Table 7. List of antibodies used with dilution factors and application (immunoblotting, immunofluorescence).

Antibody	Source	Specification
anti-E-cadherin (24E10)<br>(1:100 immunofluorescence)<br>(1 :5 000 immunoblotting)	Cell Signaling Technology	Cat# 3195
anti-EpCAM (E6V8Y)<br>(1:1 000 immunoblotting)	Cell Signaling Technology	Cat# 2929
anti-Fibronectin<br>(1:30 000 immunoblotting)	Sigma-Aldrich AB	Cat# F3648
anti-FOXA1<br>(1:1 000 immunoblotting; 4 µg ChIP)	Abcam, Cambridge, UK	Cat# ab23728
anti-SNAI1 (C15D3)<br>(1:1 000 immunoblotting)	Cell Signaling Technology	Cat# 3879
Anti-SNAI2/SLUG (C19G7)	Cell Signaling Technology	
anti-Vimentin (D21H3)<br>(1:100 immunoblotting)	Cell Signaling Technology	Cat# 5741
anti-ZEB1<br>(1:1 000 immunoblotting)	Sigma-Aldrich AB	Cat# HPA027524
anti-ZO-1<br>(1:100 immunofluorescence)	Invitrogen	Cat# 33-9100
anti-Cleaved Caspase 3 (Asp175; 5A1E)<br>(1:100 immunofluorescence)	Cell Signaling Technology	Cat# 9664
anti-HP95<br>(1:20 000 immunoblotting)	Sigma-Aldrich AB	Cat# HPA011905
anti-α-TUBULIN<br>(1:10 000 immunoblotting)	Santa-Cruz Biotechnology Inc.	Cat# sc-8035
anti-Rabbit IgG H+L) Secondary, HRP	Thermo Fisher Scientific	Cat# 65-6120
anti-Mouse IgG (H+L) Secondary, HRP	Thermo Fisher Scientific	Cat# 62-6520
anti-mouse Alexa 488 Fluor	Thermo Fisher Scientific	Cat# A21202
anti-rabbit Alexa 488 Fluor	Thermo Fisher Scientific	Cat# A21206

Supplementary Table 8: List of oligonucleotides (Fw, forward; Rev, reverse)

mRNA-specific RT-PCR primers	Sequence
SNAI1 exon1-2	Fw CTCAGATTTGACCTGTCTGCAAA<br>Rev GCTGGAAGGTAAACTCTGGATTAGA
SNAI1 exon 2	Fw ACCTCCGGAGATCCTCAAC<br>Rev CATCTGACAGGGAGGTCAG
SNAI1 exon 2-3	Fw CAAGATGCACATCCGAAGCC<br>Rev GGACAGGAGAAGGGCTTCT
TGFBRI	Fw TGGCTCAGGTTTACCATTGCTT<br>Rev AACTTCTTCTCCCCGCCACT
TGFBRII	Fw AGAGACAGTTTGCCATGACCC

	Rev ACAAGTCAGGATTGCTGGTGTT
SMAD7	Fw ACCCGATGGATTTTCTCAAACC<br>Rev GCCAGATAATTCGTTCCCCCT
SOX2	Fw ACACCCTGATCTGGCATGGA<br>Rev GGCTGTTGCCTGGCTTCTC
SOX9	Fw AAGCTCTGGAGACTTCTGAACGA<br>Rev TACTTGTAATCCGGGTGGTCCTT
ALDH1A1	Fw ATCAAAGAAGCTGCCGGGAA<br>Rev CAACAGCATTGTCCAAGTCGG
FOXA1	Fw CAGGGCTGGATGGTTGTATTG<br>Rev GTGTTCATGGAGTTCATGGAGC
ER	Fw GGGAAGTATGGCTATGGAATCTG<br>Rev TGGCTGGACACATATAGTCGTT
PGR	Fw AGCATGTCGCCTTAGAAAGTG<br>Rev TGAATCTCTGGCTTAGGGCTTG
HER2	Fw AGTGTTTGAGACTCTGGAAGAG<br>Rev TACTTGCAGGTTCTGGAAGAC
AR	Fw TATGAAGCAGGGATGACTCTG<br>Rev AGATGGGCTGACATTCATAGC
KIT	Fw AGTTCTGTGTACTCAACGTGG<br>Rev TCGCTGAACTGATAGTCAACG
CD44	Fw GGCTTTCAATAGCACCTTGC<br>Rev CAGGTCTCAAATCCGATGCT
KRT14	Fw TTGAACCTGCGCATGAGTGTG<br>Rev AGCGTCCATCTCCACATTGAC
EGFR	Fw TAGAAATCATACGCGGCAGG<br>Rev ACTTATCTCCTTGAGGGAGC
SPARC	Fw AAGAAACTGTGGCAGAGGTGAC<br>Rev ACCACCTCCTCTTCGGTTTC
GRM4	Fw TCCGAAACGTCAACTTCTCAG<br>Rev ATTGGTAGATGTCATAGCGCC
KRT8	Fw GCTGAGAGCATGTACCAGATC<br>Rev ATCCTTAATGGCCAGCTCTCC
AGR2	Fw AAGGACACAAAGGACTCTCG<br>Rev ATGTCTGAGTCCAGATGAG
**ChIP qPCR primers**	**Sequence**
*AR*_primer set 1	Fw TATTCAGGAAGCAGGGGTCC<br>Rev TTCCCACCTCCTTTTCCCTC
*AR*_primer set 2	Fw GCATACAAAGCAAACGTTTACAG<br>Rev GGAATTGCCTTTAACCATGCA
*AR*_primer set 3	Fw CCCGAGTTTGCAGAGAGGTA<br>Rev GGTTCTCTCCGCGTGCAG
GAPDH	Fw CGGCTACTAGCGGTTTTA<br>Rev AAGAAGATGCGGCTGACTGT

SUPPLEMENTARY FIGURE TITLES AND LEGENDS

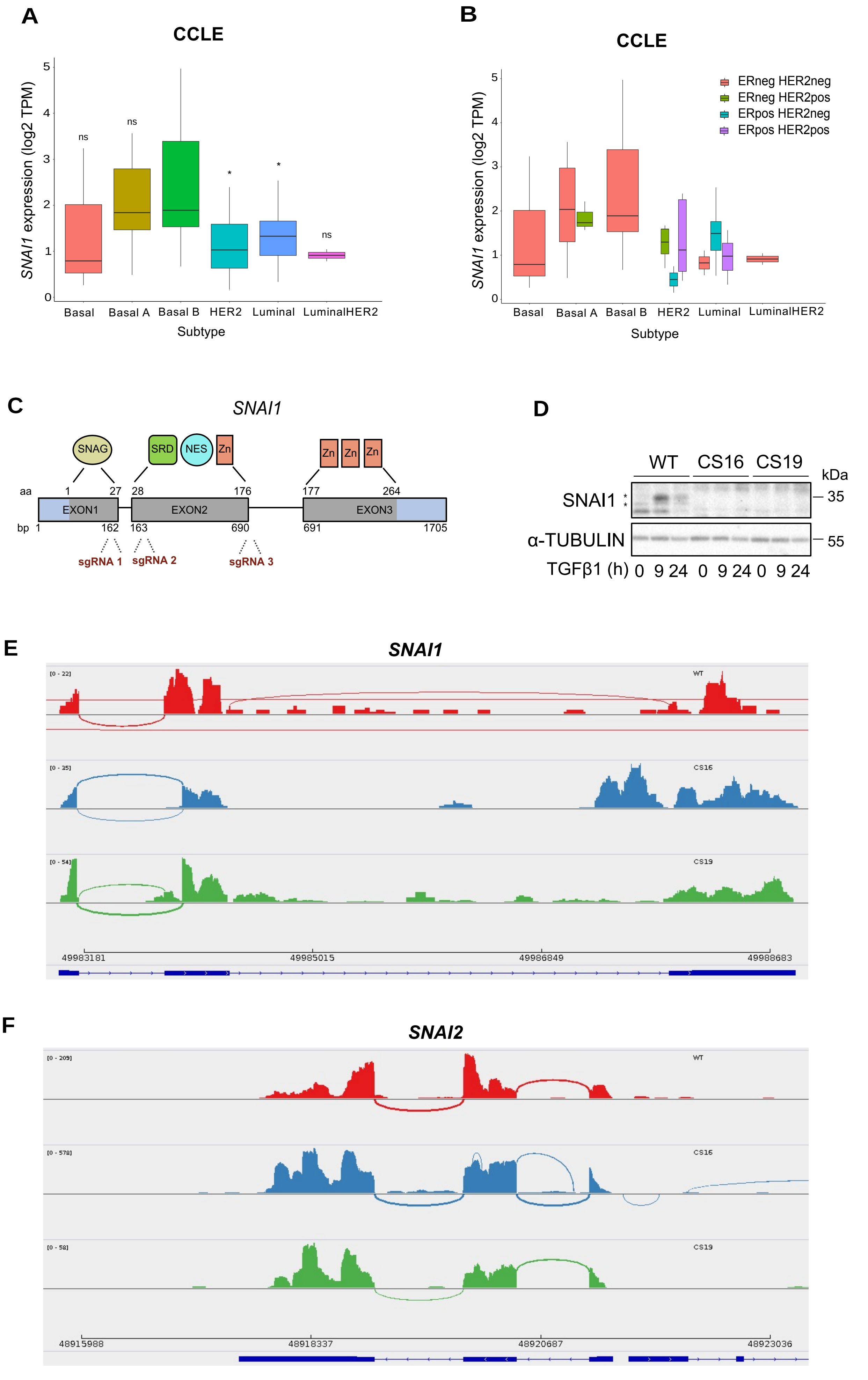

Supplementary Fig. 1

Supplementary Fig. 1: SNAI1 expression in multiple breast cancer subtypes and generation of CRISPR/Cas9-mediated *SNAI1* deletion in the Basal B, MDA-MB-231 cells.

A Expression of *SNAI1* in various subtypes of human breast cancer cell lines obtained from the CCLE portal (Basal n=3, Basal A n=15, Basal B n=11, HER2 n=13, Luminal n=15, Luminal HER2 n=2). The Basal B subtype was assigned as a reference to assess the difference in expression levels of *SNAI1* among other subtypes using the Wilcoxon rank-sum test (*U* test). **B** Expression of *SNAI1* in various subtypes of human breast cancer cell lines in the CCLE portal, based on the *ER* and *HER2* expression status (Basal n=3, Basal A n=15, Basal B n=11, HER2 n=13, Luminal n=15, Luminal HER2 n=2). Additional subtypes are indicated on the upper right. Box width reflects the number of samples. **C** Schematic representation of the human SNAI1 protein with its functional domains and the specific gRNA containing-plasmids for the CRISPR/Cas9-mediated deletion along with the corresponding three exons, number of the amino acid (aa) and base pair (bp) residues. SNAG, regulatory domain; SRD, serine-rich domain; NES, nuclear export signal; Zn, zinc-finger structural motifs. Untranslated regions are presented with blue color. **D** Representative immunoblot of three biological replicates along with molecular mass markers in kDa showing the expression levels of SNAI1 in MDA-MB-231-WT and *SNAI1*-KO cells stimulated with 5 ng/ml TGFβ1 for the indicated time periods. α-Tubulin serves as loading control. Stars indicate the specific protein bands. **E, F** Sashimi plot visualization of aligned RNA-sequencing reads obtained from MDA-MB-231-WT and *SNAI1*-KO cells against *SNAI1* and *SNAI2* exons of the reference genome, with corresponding base pair coordinates and a bottom illustration of the exon (blue boxes) and intron (lines) organization of the genes.

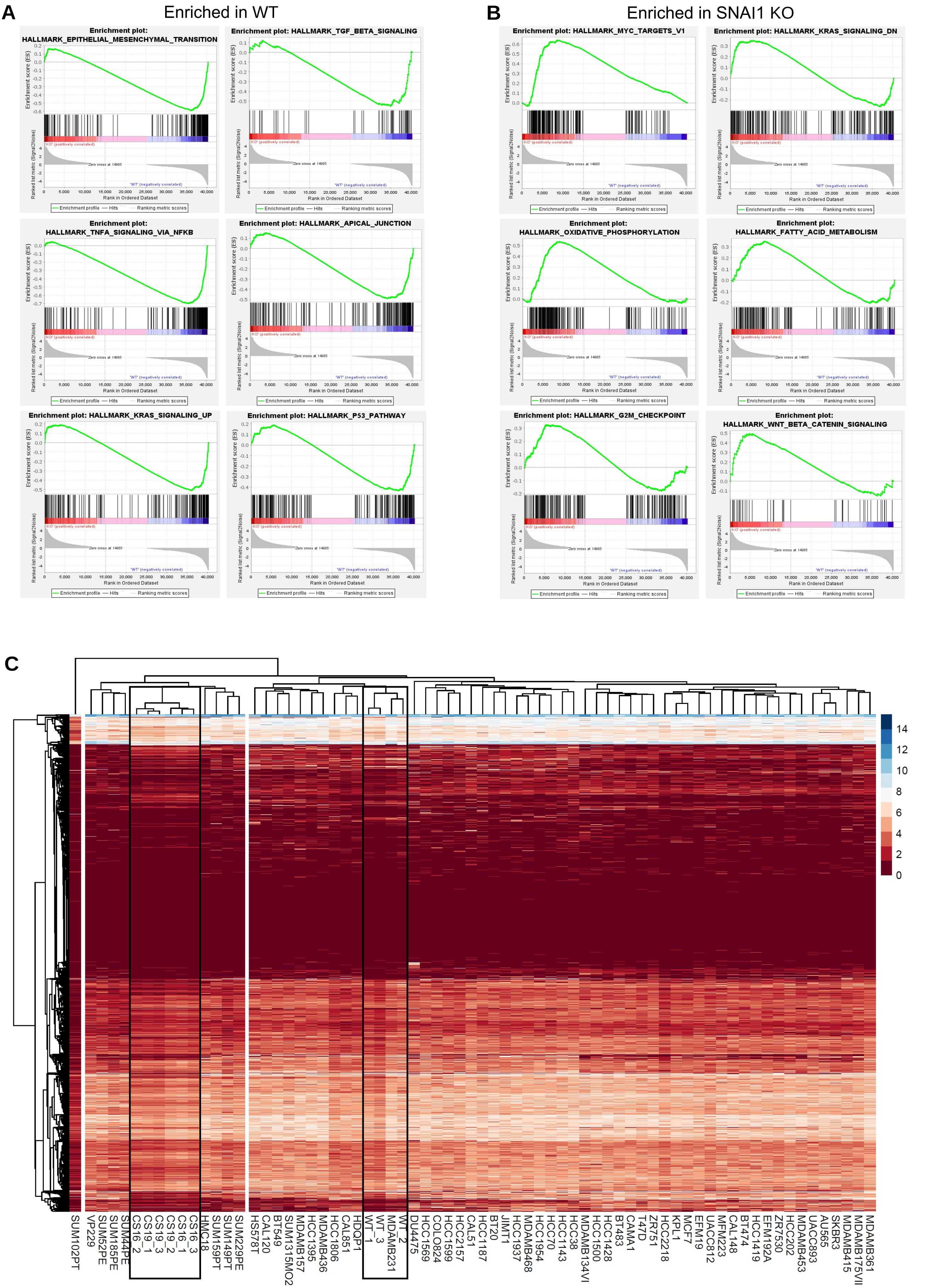

Supplementary Fig. 2

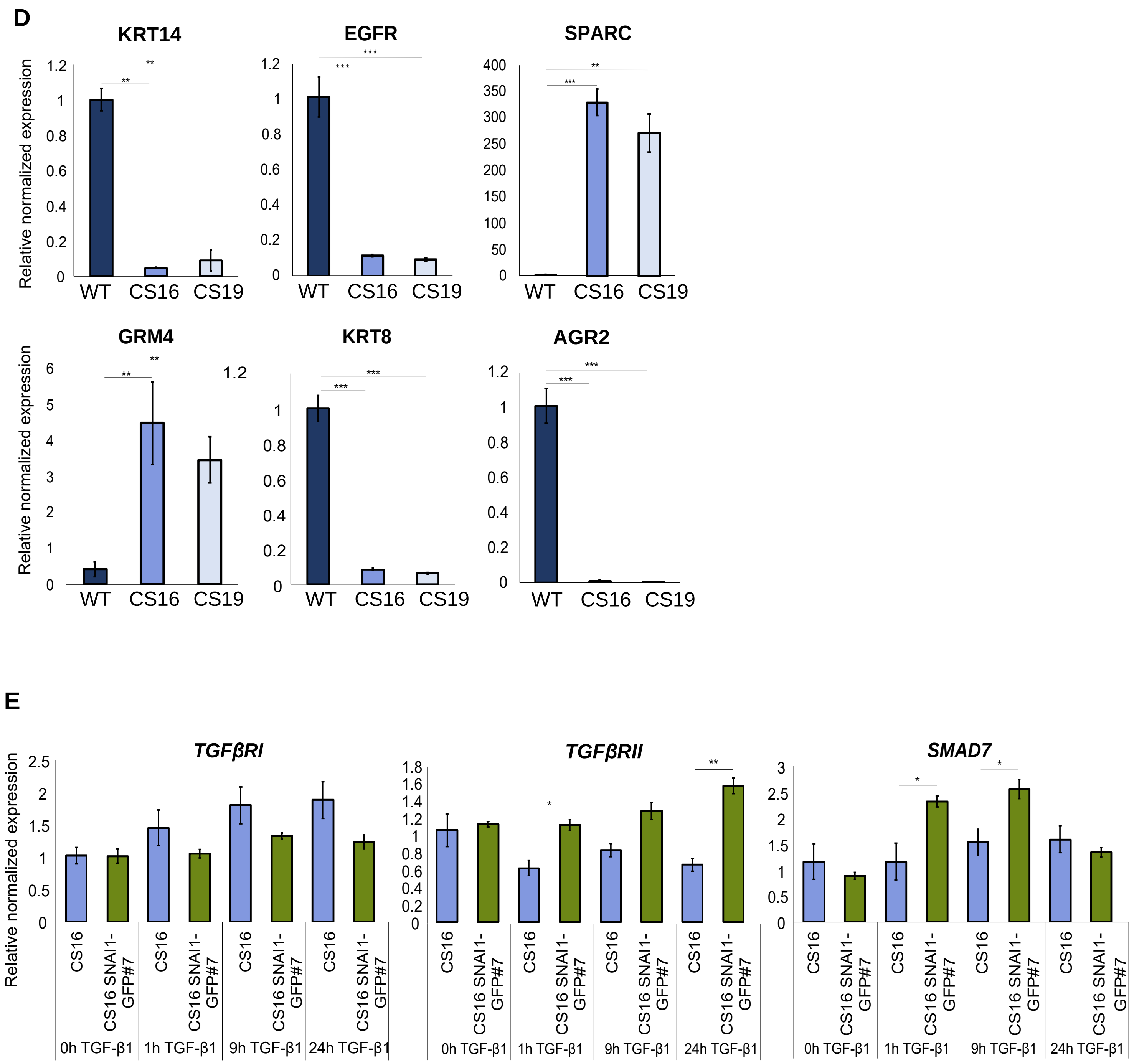

Supplementary Fig. 2

Supplementary Fig. 2: Transcriptomic analysis of MDA-MB-231-WT and *SNAI1* knockout cells and acquisition of an epithelio-mesenchymal phenotype.

A, B Enrichment plots of the top significantly enriched hallmarks obtained from gene set enrichment analysis of MDA-MB-231-WT **(A)** and *SNAI1*-KOs **(B)**. **C** Heatmap illustrating the hierarchical clustering based on RNA expression profiles of 18 817 genes in 61 breast cancer cell lines obtained from the CCLE portal, including the reference MDA-MB-231 cell line, in addition to triplicate (1-3) samples of our *SNAI1*-KOs (CS16 and CS19) and MDA-MB-231-WT cells. The color-coded scale represents the transformed values ($\log_2$+1) of normalized transcript per kilobase million (TPM) quantified reads. **D** RT-qPCR analysis of the basal *KRT14*, *EGFR*, *SPARC* and luminal *GRM4*, *KRT8*, *AGR2* mRNA levels in MDA-MB-231-WT and *SNAI1*-KO cells. **E** RT-qPCR analysis of *TGFBRI*, *TGFBRII* and *SMAD7* mRNA levels in CS16 *SNAI1*-KOs and CS16 SNAI1-GFP#7 cells stimulated with 5 ng/ml of TGFβ1 for the indicated time periods. Values in **D** and **E** represent fold-change of mRNA expression normalized to *GAPDH*. Data in **D** and **E** are presented as mean values of three biological replicates ± SEM, each in technical triplicates and p-values are shown based on two-tailed unpaired Student's *t*-test. p-values *p≤ 0.05, **p≤ 0.01, ***p≤ 0.001.

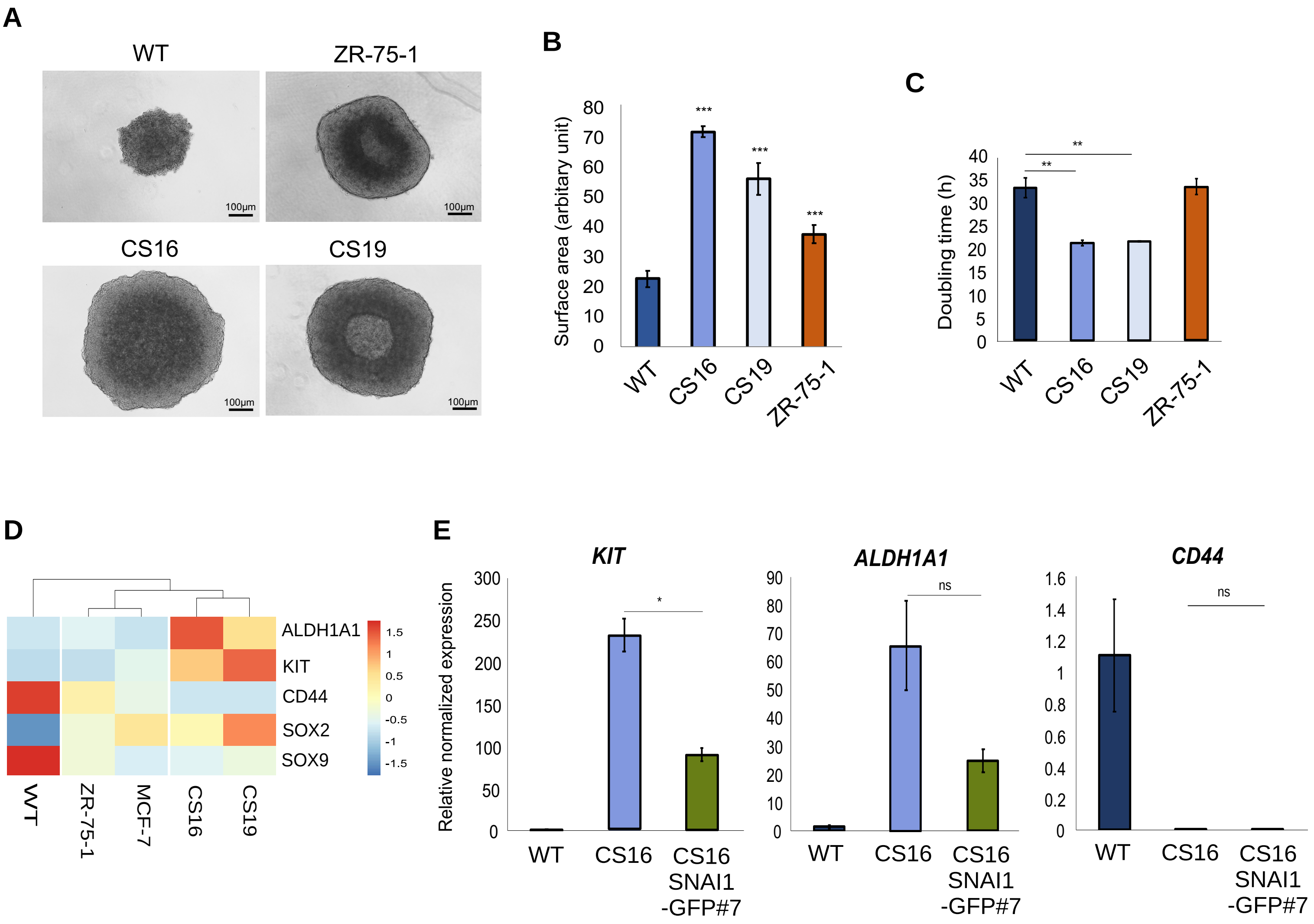

Supplementary Fig. 3

Supplementary Fig. 3: *SNAI1* knockout cells obtain high proliferative and epithelial stem/progenitor-like traits.

A Representative phase contrast images from three biological repeats showing the morphology of the mammospheres derived from MDA-MB-231-WT, *SNAI1*-KOs and ZR-75-1. Scale bars=100 µm. **B** Quantification of the cross-section area of the mammospheres. **C** Proliferation of MDA-MB-231-WT, *SNAI1*-KOs and ZR-75-1 cells. Equal number of cells was initially seeded, and viable cell number was counted at day 5. **D** Heat map representing the mean mRNA expression levels of stem cell-associated genes measured by RT-qPCR analysis in three independent biological replicates of ZR-75-1, MCF-7, MDA-MB-231-WT and *SNAI1*-KO cells. mRNA expression was normalized to *GAPDH*. The hierarchical clustering of ZR-75-1, MCF-7, MDA-MB-231-WT and *SNAI1*-KOs was performed using Euclidean distance based on the expression of the indicated genes. **E** RT-qPCR analysis of *c-KIT*, *ALDH1A1* and *CD44* in MDA-MB-231-WT, CS16 and CS16 SNAI1-GFP#7. Values represent fold-change of mRNA expression normalized to *GAPDH*. Data in **B**, **C** and **E** are presented as mean values of three biological replicates ± SEM, each in technical triplicates and p-values are shown based on two-tailed unpaired Student's *t*-test. p-values $^{*}p \leq 0.05$, $^{**}p \leq 0.01$, $^{***}p \leq 0.001$.

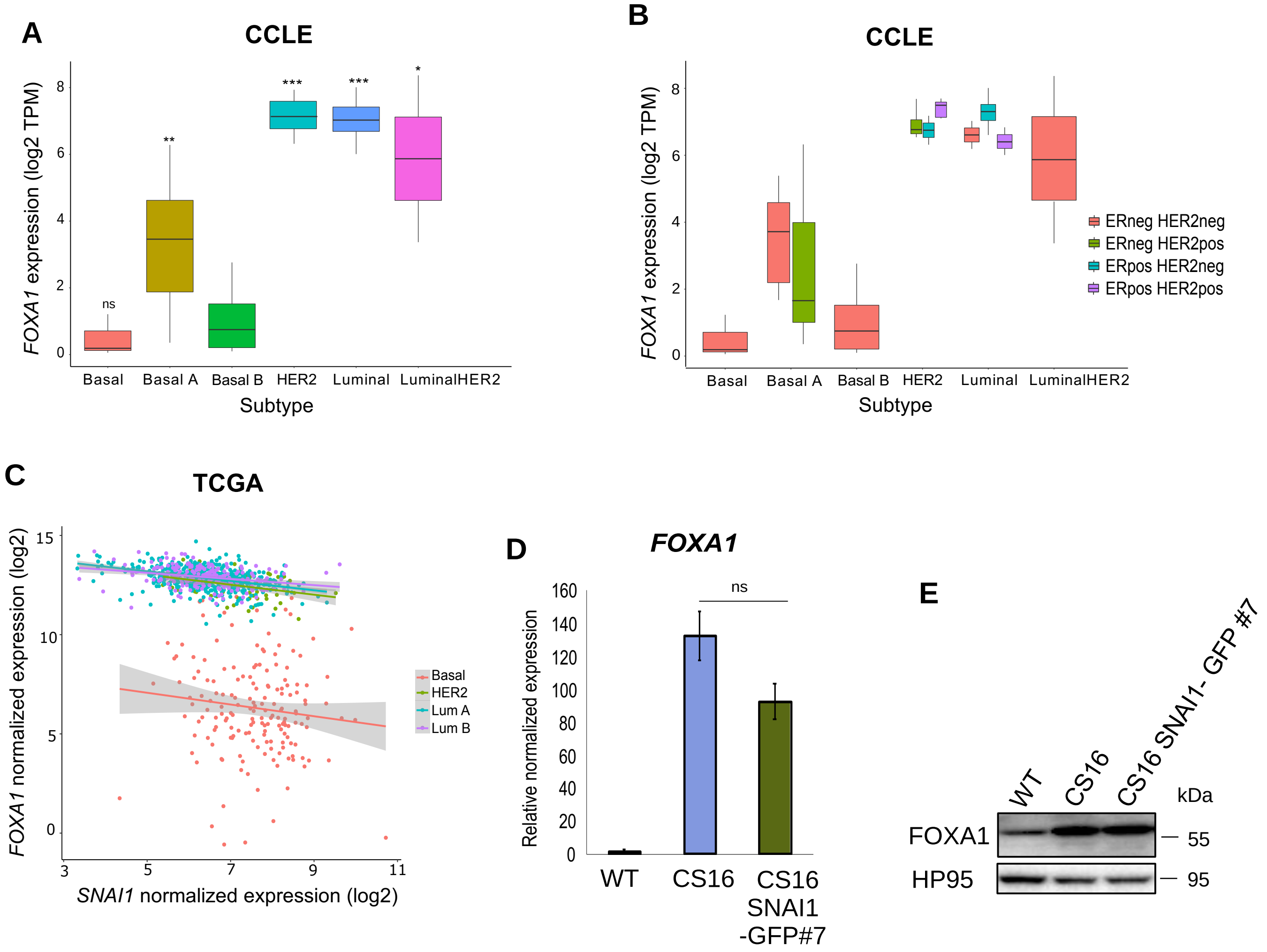

F

Regression Statistics (FOXA1/SNAI1)				
Multiple R	0.38800533			
R Square	0.150548136			
Adjusted R Square	**0.147697627**			
Standard Error	0.928095839			
	Coefficients	*Standard Error*	*t Stat*	*P-value*
Intercept	8.247239218	0.14578	56.57338	2E-161
FOXA1(1+log2)	-0.108628065	0.014947	-7.26736	**3.23E-12**

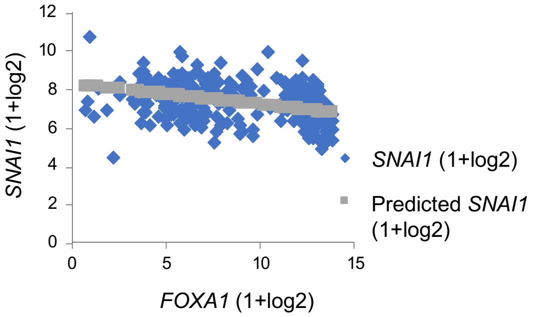

Supplementary Fig. 4

Supplementary Fig. 4: *SNAI1* expression is inversely correlated to *FOXA1* expression.

A Expression of *FOXA1* in various subtypes of human breast cancer cell lines in the CCLE portal (Basal n=3, Basal A n=15, Basal B n=11, HER2 n=13, Luminal n=15, Luminal HER2 n=2). The difference in expression levels was assessed in comparison to Basal B subtype using Wilcoxon rank-sum test (*U* test). **B** Expression of *FOXA1* in CCLE breast cancer cells separated by *ER* and *HER2* expression status. Additional subtypes are indicated on the upper right. Box width reflects the number of samples. **C** Scatter plot showing the correlation between *SNAI1* and *FOXA1* in TCGA breast cancer samples color-coded according to the molecular subtype. **D** RT-qPCR analysis of *FOXA1* in MDA-MB-231-WT, CS16 and CS16 SNAI1-GFP#7 cells. Values represent fold-change of mRNA expression normalized to *GAPDH*. Data are presented as mean values of three biological replicates ± SEM, each in technical triplicates and p-values are shown based on two-tailed unpaired Student's *t*-test. **E** Representative immunoblot of three biological replicates showing the protein expression levels of FOXA1 in MDA-MB-231-WT, CS16 and CS16 SNAI1-GFP#7 cells. HP95 serves as loading control. **F** Regression analysis of *FOXA1* and *SNAI1* expression in TCGA breast cancer samples demonstrating the observed versus the predicted estimation of *SNAI1* expression using *FOXA1* as a predictor variant. The statistical significance was derived using ANOVA test. p-values *$p \leq 0.05$, **$p \leq 0.01$, ***$p \leq 0.001$.

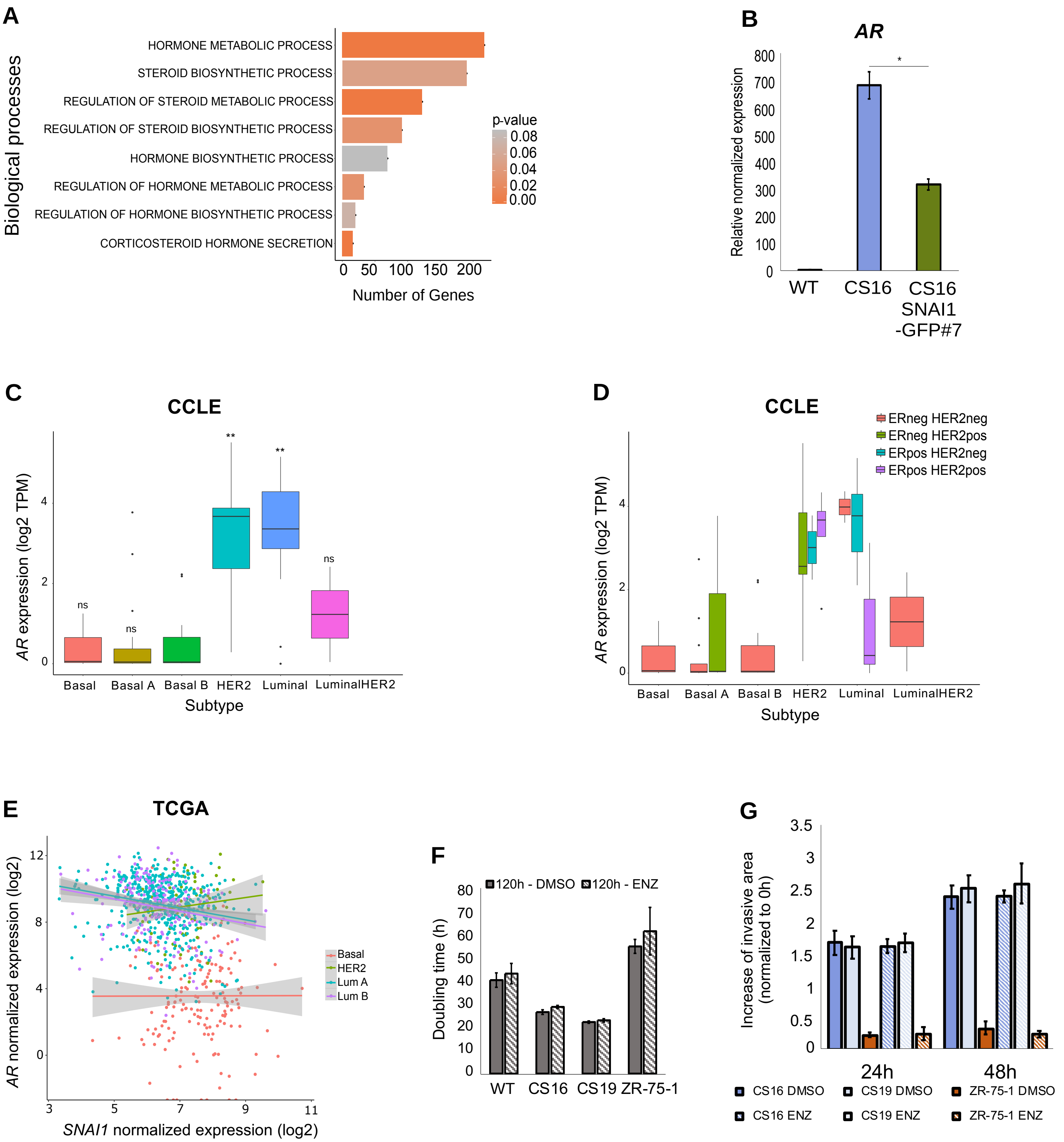

Supplementary Fig. 5

H

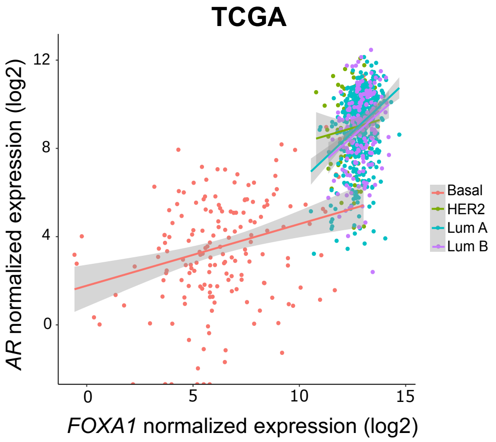

I

Regression Statistics (FOXA1/AR)	
Multiple R	0.795019859
R Square	0.632056575
Adjusted R Square	**0.630821866**
Standard Error	1.976289345

	Coefficients	Standard Error	t Stat	P-value
Intercept	-0.550734441	0.310423201	-1.77414	0.077061
FOXA1(1+log2)	0.72014325	0.031829023	22.62536	**1.16E-66**

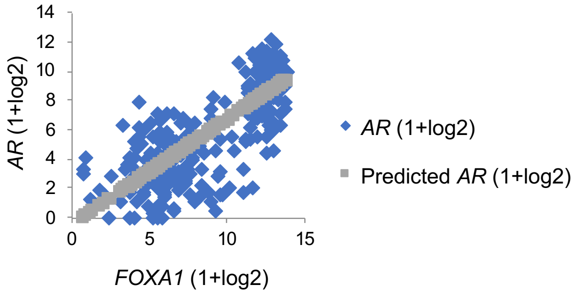

J

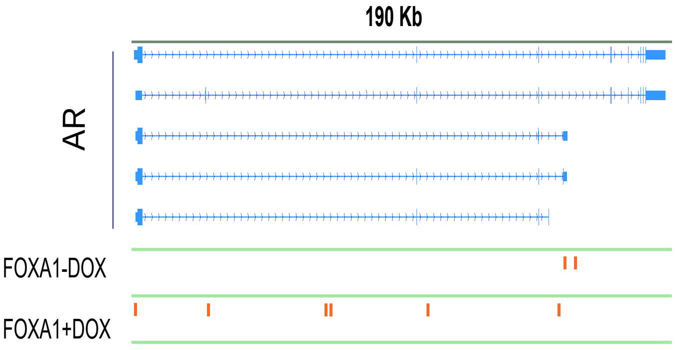

K

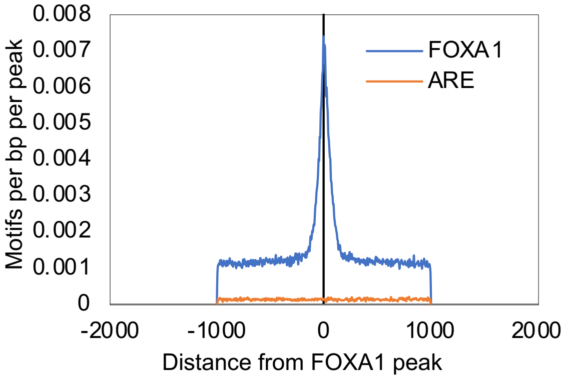

Supplementary Fig. 5

Supplementary Fig. 5: Upon *SNAI1* knockout, FOXA1 shifts the differentiation balance of MDA-MB-231 cells toward a non-basal, luminal-like state by regulating AR expression.

A Bar graph showing the biological processes relevant to hormone biosynthesis/metabolism enriched in *SNAI1*-KO cells. The horizontal axis indicates the number of genes annotated to each process and the color code represent the corresponding p-value. **B** RT-qPCR analysis of *AR* in MDA-MB-231-WT, CS16 and CS16 SNAI1-GFP#7 cells. Values represent fold-change of mRNA expression normalized to *GAPDH*. Data are presented as mean values of three biological replicates ± SEM, each in technical triplicates and p-values are shown based on two-tailed unpaired Student's *t*-test. **C** Expression of *AR* in various subtypes of human breast cancer cell lines obtained from the CCLE portal (Basal n=3, Basal A n=15, Basal B n=11, HER2 n=13, Luminal n=15, Luminal HER2 n=2). The difference in expression levels was assessed in comparison to Basal B subtype using Wilcoxon rank-sum test (*U* test). **D** Expression of *AR* in the same CCLE breast cancer cells separated by *ER* and *HER2* expression status. Additional subtypes are indicated on the upper right. Box width reflects the number of samples. **E** Scatter plot demonstrating the correlation between *SNAI1* and *AR* in breast cancer samples obtained from TCGA dataset. Breast cancer samples are color-coded based on the corresponding subtypes. **F** Proliferation of *SNAI1*-KOs and ZR-75-1 cells in the presence of either DMSO or 0.05 μM enzalutamide. Equal number of cells was initially seeded, and viable cell number was counted at day 5. **G** Quantification of the invasion area subtracted by the mammosphere area of *SNAI1*-KO and ZR-75-1 mammospheres embedded in collagen after 24 or 48 h in the presence of either DMSO or 0.05 μM enzalutamide. Data in **F** and **G** are presented as mean values ± SEM of three biological replicates, each in technical triplicates and p-values are shown based on two-tailed unpaired Student's *t*-test. **H** Scatter plot showing the correlation between *FOXA1* and *AR* in TCGA breast cancer samples color-coded according to the

molecular subtype. **I** Regression analysis of *FOXA1* and *AR* expression in TCGA breast cancer samples demonstrating the observed versus the predicted estimation of *AR* expression using *FOXA1* as a predictor variant. The statistical significance was derived using ANOVA test. J FOXA1 binding to the *AR* transcriptional start sites upon FOXA1 overexpression (+Dox) and in the absence of FOXA1 induction (-Dox) in MCF-7 BRCA cells. The five *AR* transcript isoforms are shown in blue, while the orange lines depict the multiple binding sites of FOXA1 (-Dox, +Dox) to *AR*. **K** Cumulative histogram showing the enrichment of FOXA1 or *androgen responsive elements* (*ARE*) over the target regions identified by FOXA1 ChIP-sequencing analysis in MCF-7 cells.

A

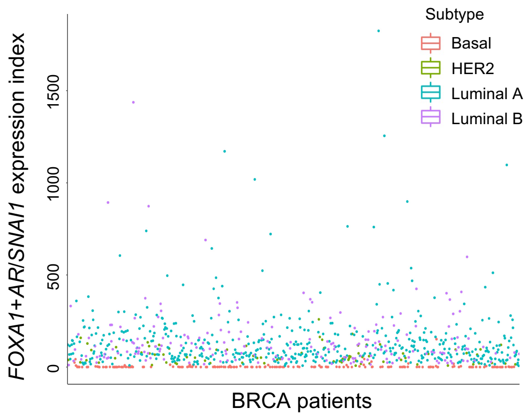

Median values of Index	Basal	HER2	Luminal A	Luminal B
	13.11	144.51	434.98	848.00

B

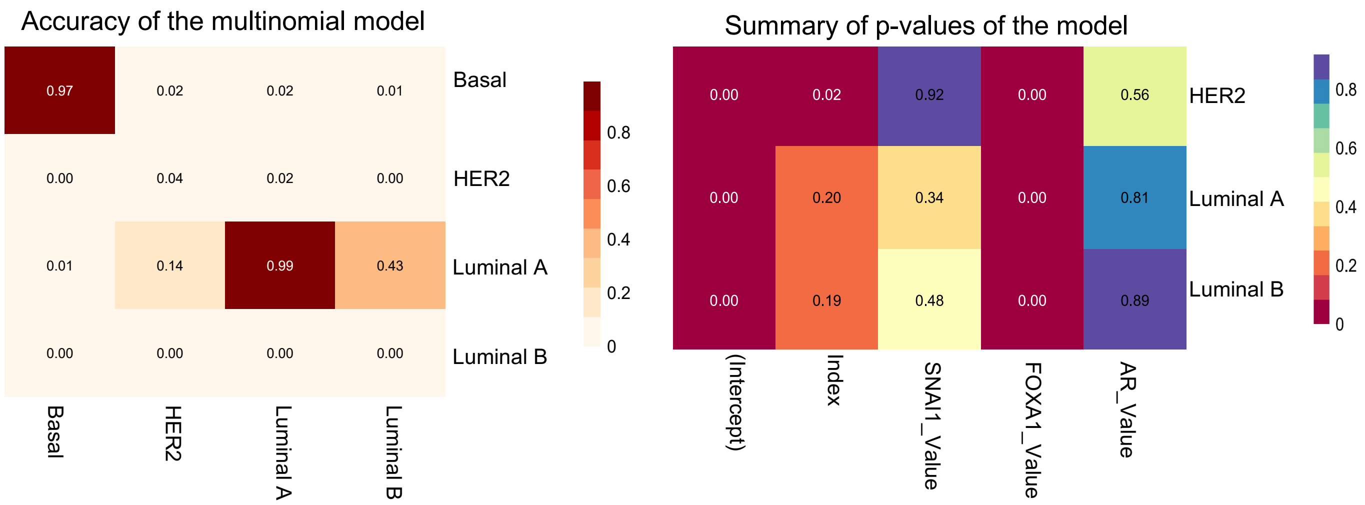

C

Summary of the binomial regression model

	Estimate	Std.Error	Z value	p-value(>|Z|)
Intercept	-4.3788029	0.5695279	-7.688	1.49E-14
FOXA1	0.0019796	0.0002154	9.192	2.00E-16

Null deviance: 893.66 on 944 degrees of freedom
Residual deviance: 87.13 on 943 degrees of freedom

ANOVA statistics of the model

	Df	Deviance	Resid. Df	Resid. Dev	P-value(>Chi)
Null			944	893.66	
FOXA1	1	806.53	943	87.13	2.20E-16

D

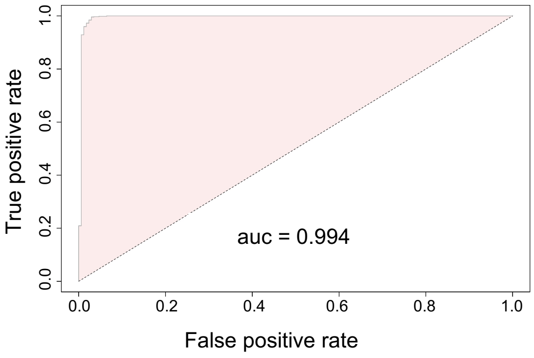

Supplementary Fig. 6

Supplementary Fig. 6 Predictive value of the SNAI1/FOXA1/AR transcriptional network in breast cancer subtypes.

A Scatter plot showing the distribution of the combined (*FOXA1+AR*)/*SNAI1* expression index among TCGA breast cancer samples. The samples are color-coded according to the subtype. **B** Heatmap illustrating the p-values associated with each variable in the multinomial model. **C** Confusion matrix summarizing the accuracy of the predictive multinomial model in predicting the correct subtype of TCGA breast cancer tissues. **D** ROC showing the false positive rate versus the true positive rates of the *FOXA1* predictive binomial model in discriminating between TCGA basal and non-basal breast cancer samples.

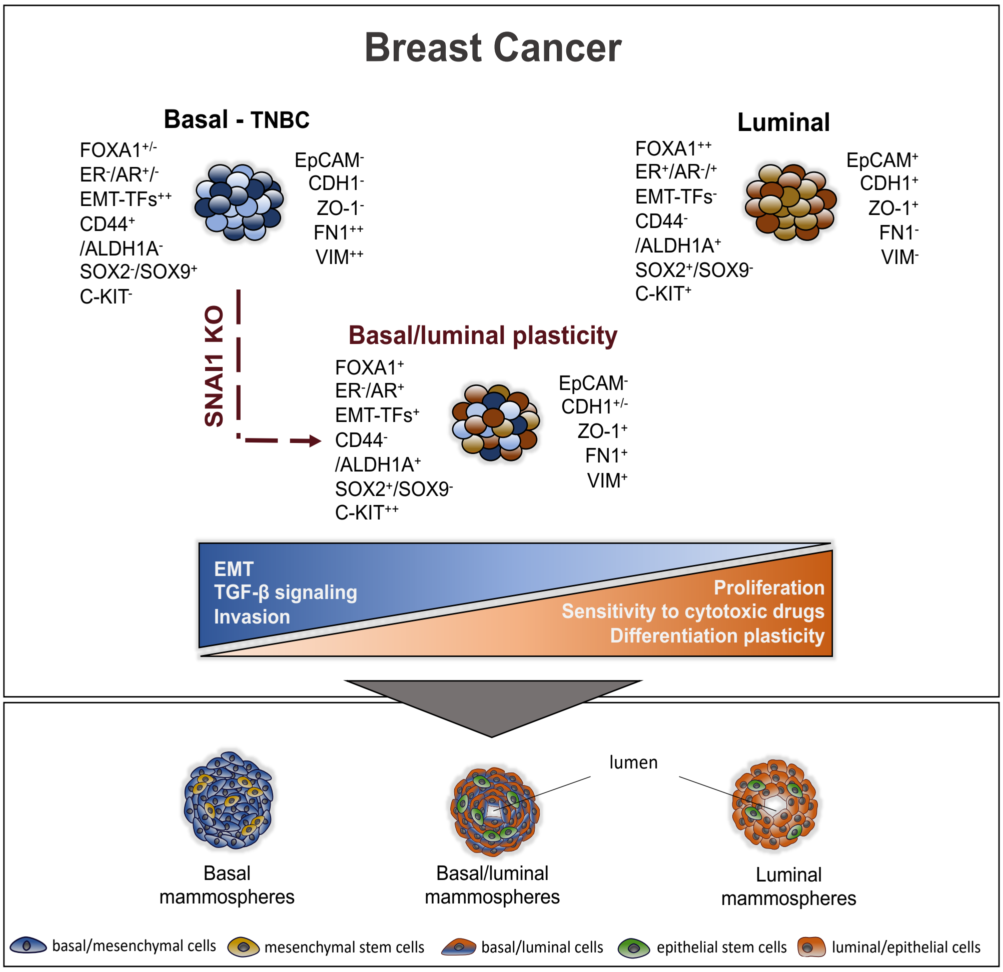

Supplementary Fig. 7

Supplementary Fig. 7 Proposed molecular model of cellular heterogeneity upon *SNAI1* knockout in breast cancer.

Basal-TNBC and luminal breast cancer subtypes are illustrated as aggregates of heterogeneous cell populations of different colors along with genes that define the identity of each subtype. Starting from the basal-TNBC, knockout of *SNAI1* (dotted arrow) generates a basal/luminal intermediate phenotype demonstrating differentiation plasticity. Specific biological functions are listed inside triangles colored in the same way as the breast cancer subtypes. Each triangle represents a gradient of biological function from maximal to minimal. Illustrations of the corresponding mammospheres generated by the subtypes shown on top are listed at the bottom along with an explanation of specific cell types that assemble the mammospheres. The basal/luminal cell phenotype in the central axis of the figure is a new contribution derived from this study.

Source data main Figure 1E

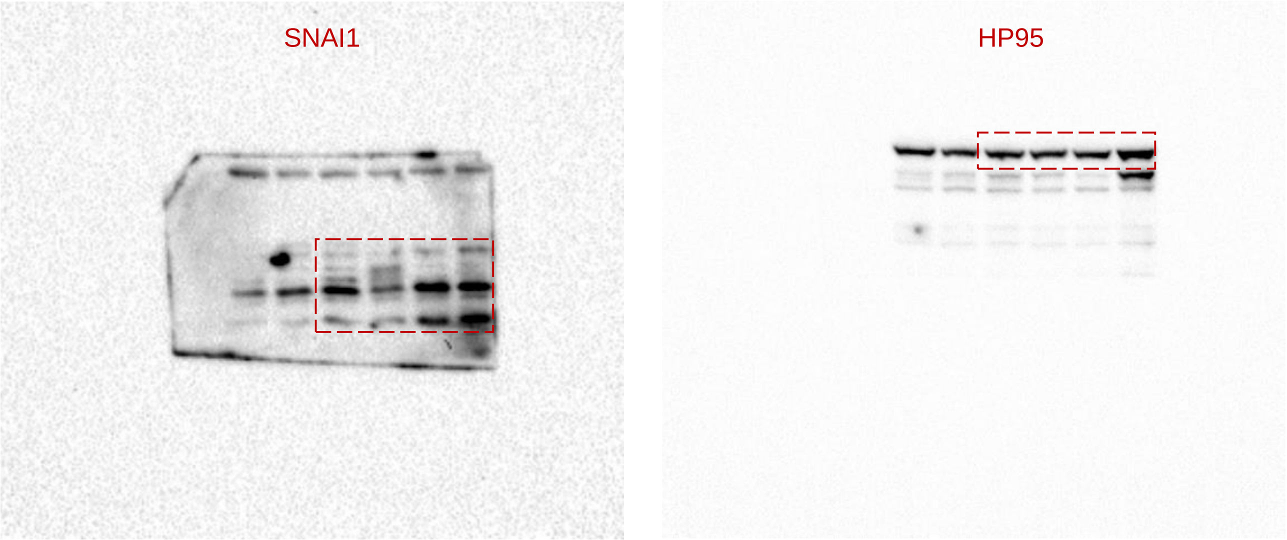

Source data main Figure 1F

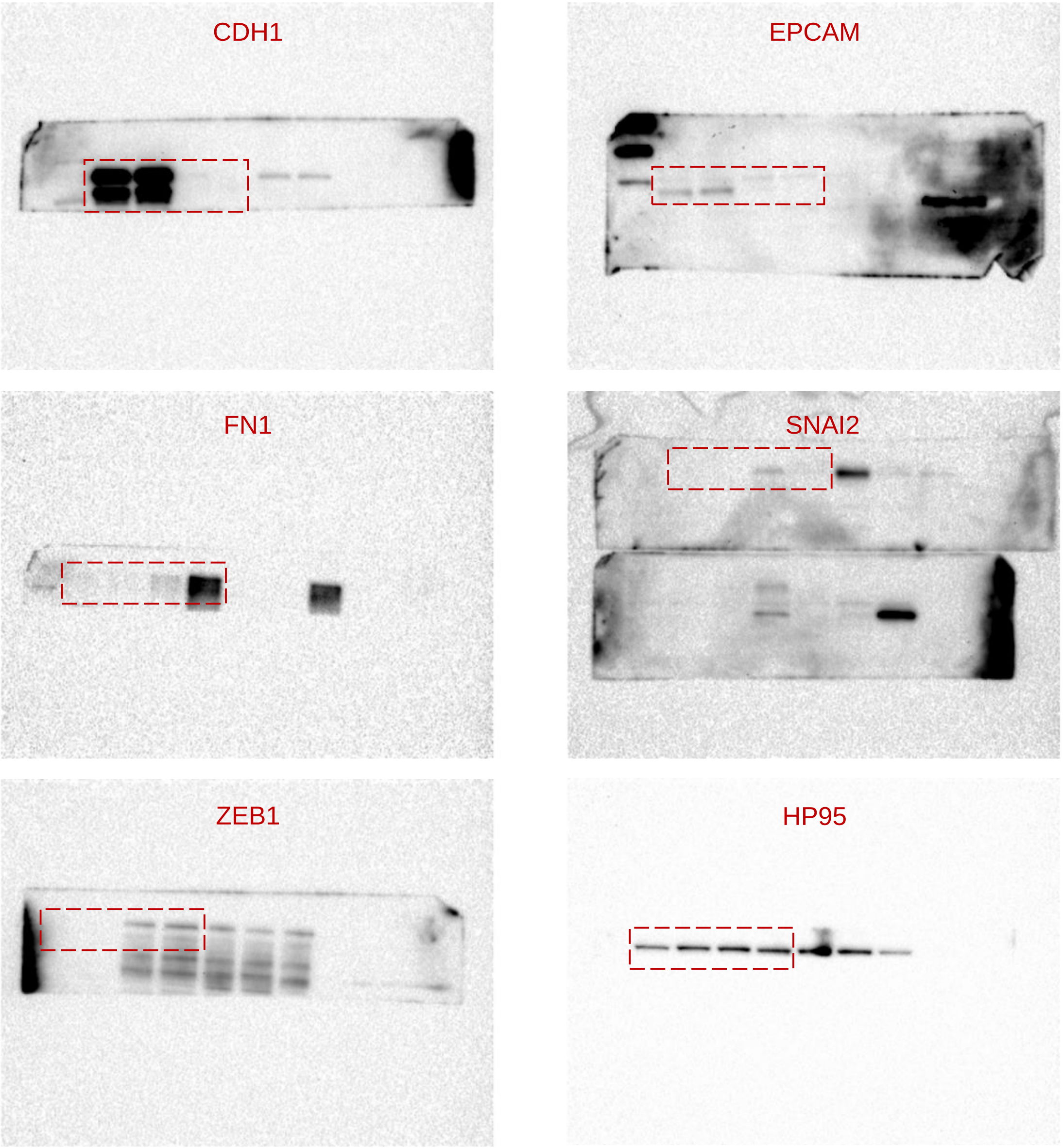

Source data main Figure 2G

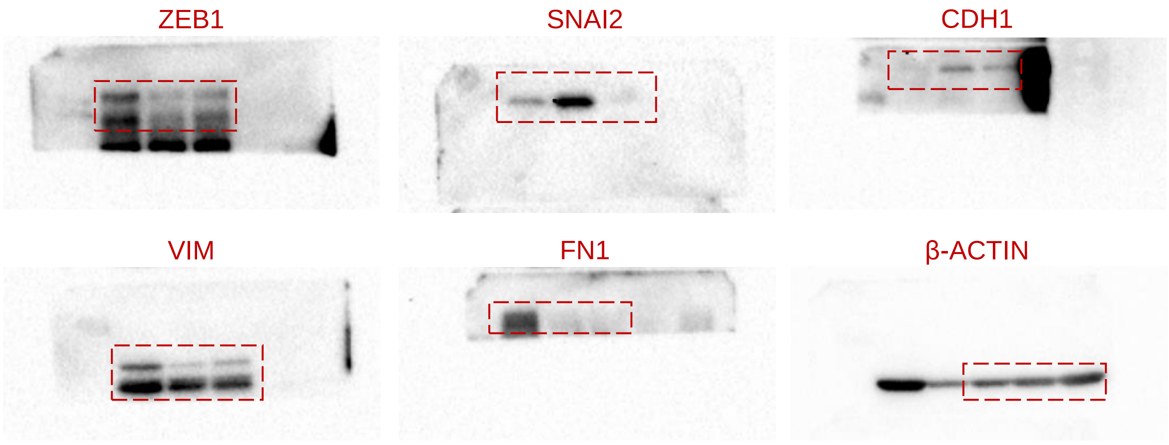

Source data main Figure 2H

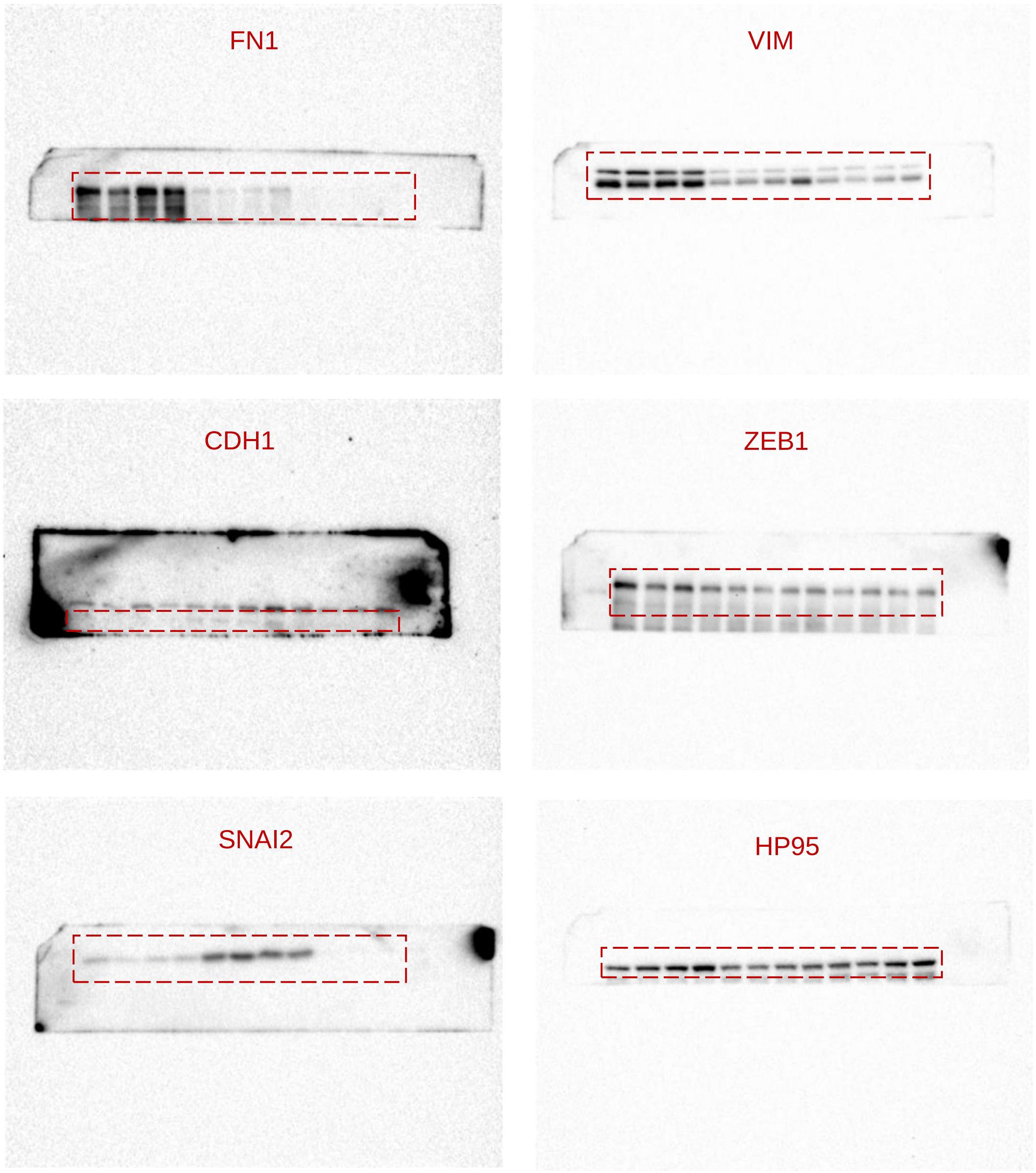

Supplementary Fig. 8

Source data main Figure 3B

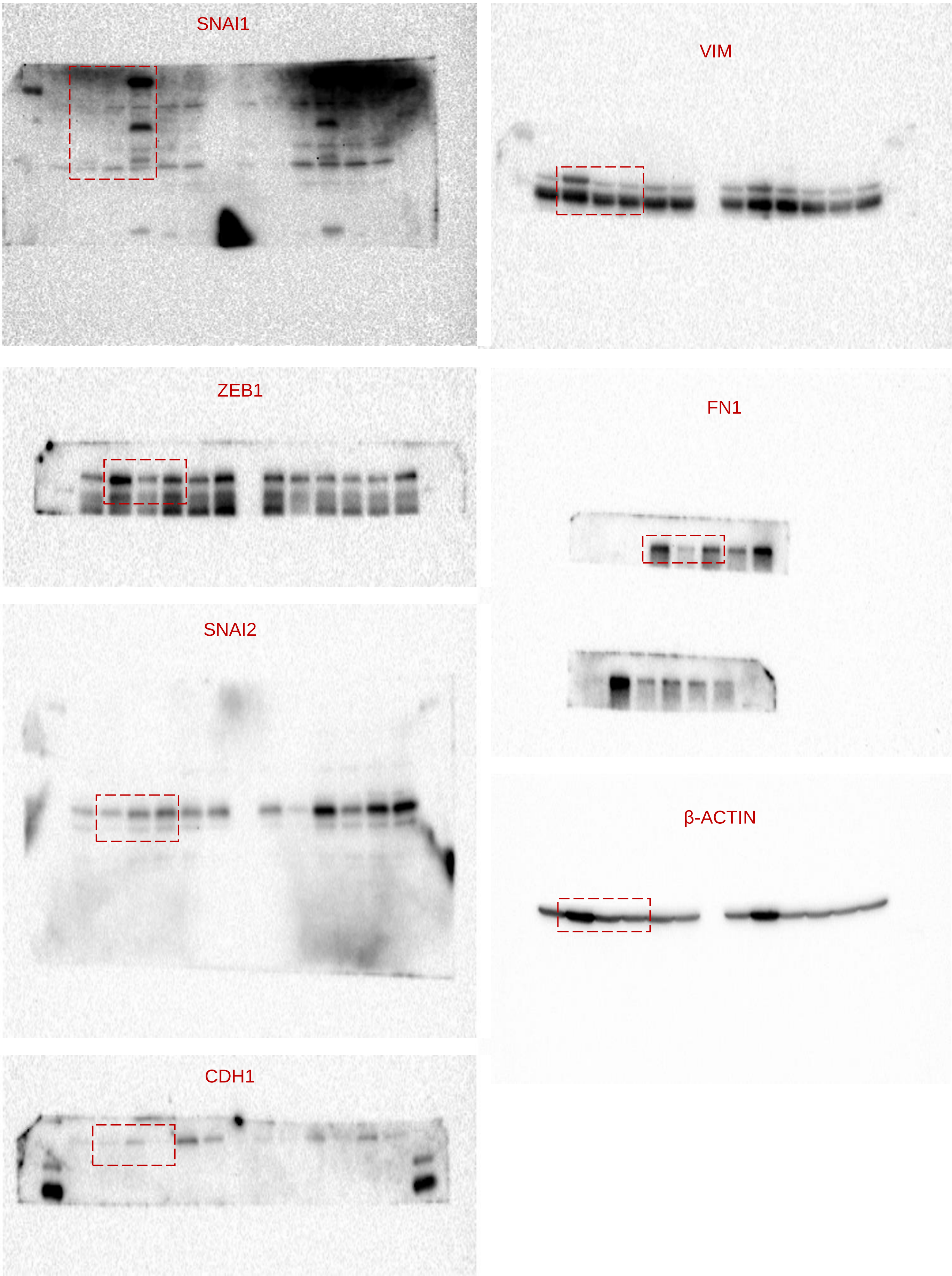

Supplementary Fig. 8

Source data main Figure 3C

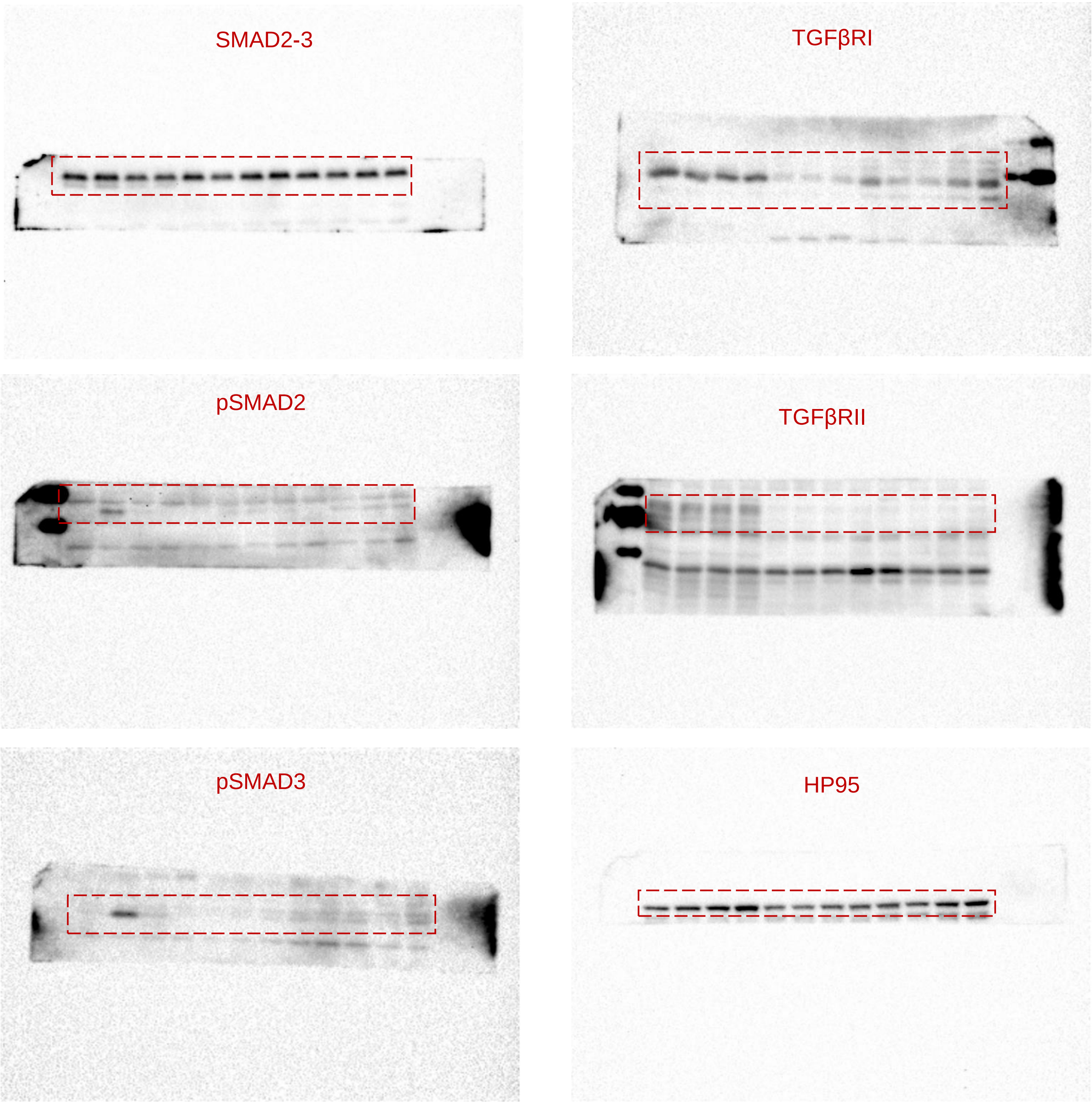

Source data main Figure 3E

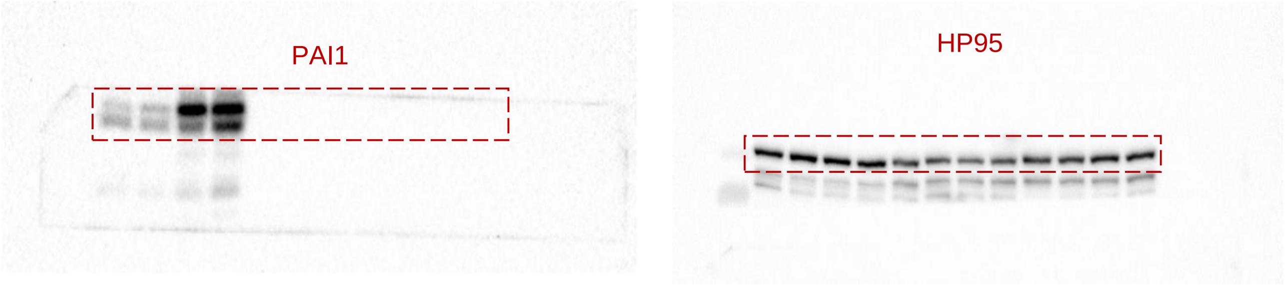

Source data main Figure 6E

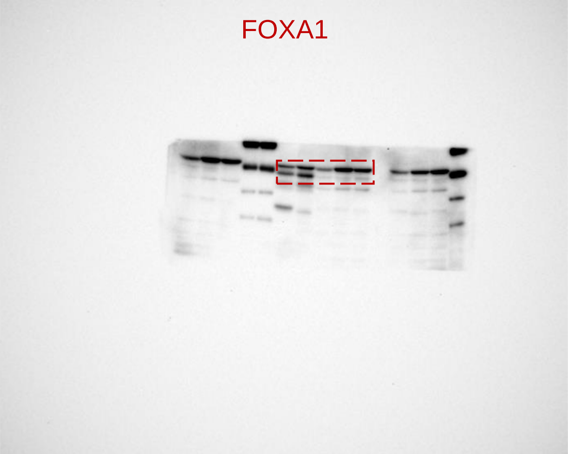

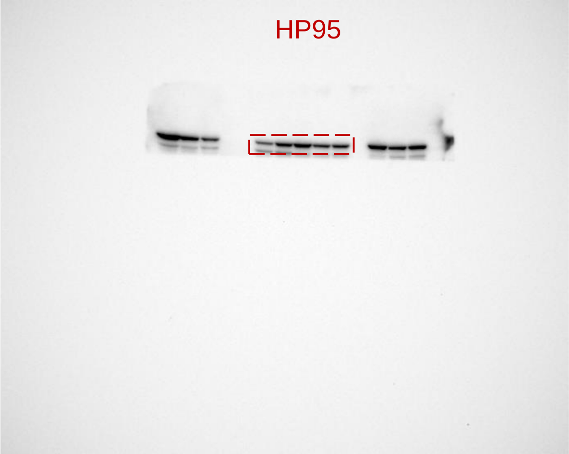

Supplementary Fig. 8

Source data supplementary Figure 1D

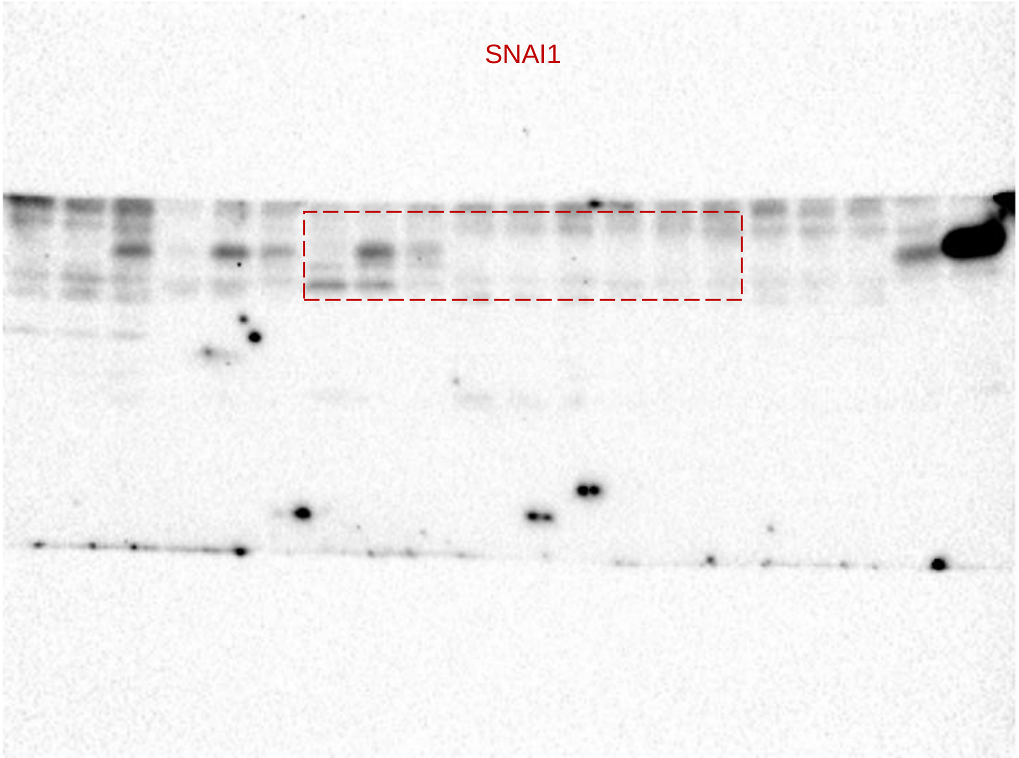

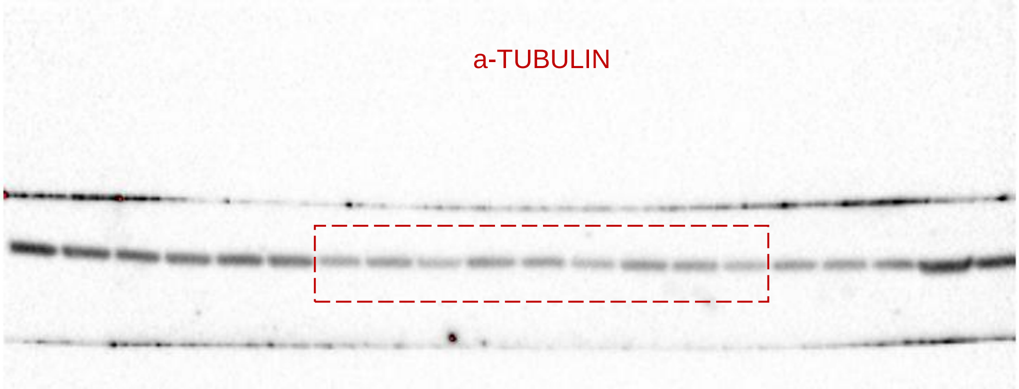

Source data supplementary Figure 4E

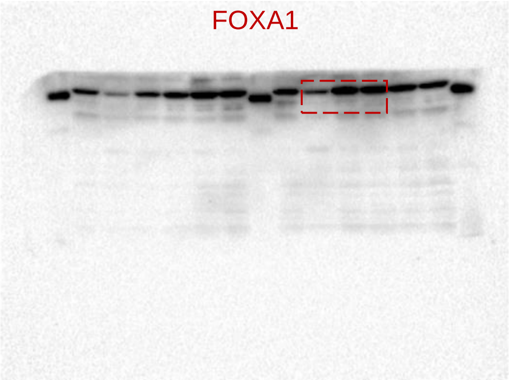

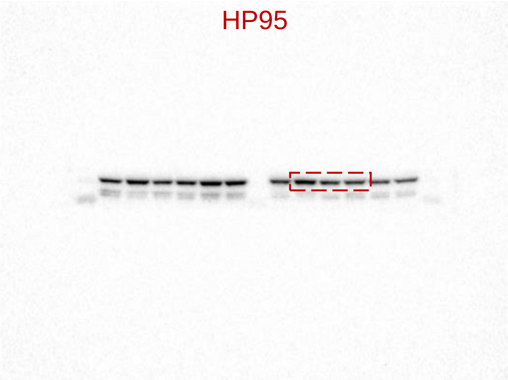

Supplementary Fig. 8: Original immunoblots.

A series of original immunoblots with dotted rectangles marking the area of the immunoblot that was cropped and placed in the main or supplementary figures, along with reference to each of the corresponding figures.
